# Nuclear Accumulation of LAP1:TRF2 Complex during DNA Damage Response Uncovers a Novel Role for LAP1

**DOI:** 10.3390/cells9081804

**Published:** 2020-07-29

**Authors:** Cátia D. Pereira, Filipa Martins, Mariana Santos, Thorsten Müeller, Odete A. B. da Cruz e Silva, Sandra Rebelo

**Affiliations:** 1 Neuroscience and Signaling Laboratory, Institute of Biomedicine (iBiMED), Department of Medical Sciences, University of Aveiro, 3810-193 Aveiro, Portugal; daniela.pereira@ua.pt (C.D.P.); samartins@ua.pt (F.M.); marianasmg@gmail.com (M.S.); odetecs@ua.pt (O.A.B.d.C.eS.); 2 Cell Signaling in Neurodegeneration (CSIN), Medical Proteome-Center, Ruhr-University Bochum, 44801 Bochum, Germany; thorsten.t.mueller@ruhr-uni-bochum.de

**Keywords:** LAP1, TRF2, γ-H2AX, DNA damage, DNA repair, hydrogen peroxide, bleomycin, nuclear envelope, nucleoplasm, protein phosphorylation

## Abstract

Lamina-associated polypeptide 1 (LAP1) is a nuclear envelope (NE) protein whose function remains poorly characterized. In a recent LAP1 protein interactome study, a putative regulatory role in the DNA damage response (DDR) has emerged and telomeric repeat-binding factor 2 (TRF2), a protein intimately associated with this signaling pathway, was among the list of LAP1 interactors. To gain insights into LAP1′s physiological properties, the interaction with TRF2 in human cells exposed to DNA-damaging agents was investigated. The direct LAP1:TRF2 binding was validated in vitro by blot overlay and in vivo by co-immunoprecipitation after hydrogen peroxide and bleomycin treatments. The regulation of this protein interaction by LAP1 phosphorylation was demonstrated by co-immunoprecipitation and mass spectrometry following okadaic acid exposure. The involvement of LAP1 and TRF2 in the DDR was confirmed by their increased nuclear protein levels after bleomycin treatment, evaluated by immunoblotting, as well as by their co-localization with DDR factors at the NE and within the nucleoplasm, assessed by immunocytochemistry. Effectively, we showed that the LAP1:TRF2 complex is established during a cellular response against DNA damage. This work proposes a novel functional role for LAP1 in the DDR, revealing a potential biological mechanism that may be disrupted in LAP1-associated pathologies.

## 1. Introduction

The nuclear envelope (NE) is a highly organized and dynamic double membrane that extends from the endoplasmic reticulum (ER) and surrounds the eukaryotic nucleus. In terms of its general architecture, the NE is formed by the inner (INM) and outer (ONM) nuclear membranes, which are two contiguous lipid bilayers harboring specific transmembrane proteins that face, respectively, the nuclear interior and the cytoplasm, being separated by a narrow perinuclear space (PNS). Furthermore, the NE contains large multiprotein nuclear pore complexes (NPCs) working as transport channels at sites where the nuclear membranes are physically interconnected. In metazoans, the NE is additionally composed of the nuclear lamina, a fibrous network juxtaposed to the INM that consists primarily of type V intermediate filaments known as A- and B-type lamins [1]. From a functional point of view, besides providing mechanical stability to the nucleus and acting as a protective barrier for the genome, the NE regulates pivotal nuclear processes (e.g., spatial genome organization, gene expression, DNA replication and repair) in virtue of the ability of lamins [2], as well as INM [3,4] and NPC [5,6] proteins, to bind DNA, histones and/or chromatin-modifying factors. The fact that the NE is of paramount importance for the maintenance of cellular homeostasis has been a matter of interest for researchers, especially considering that numerous diseases are causatively linked to mutations in NE protein-encoding genes [7,8]. In particular, genetic alterations in the human *TOR1AIP1* gene, which encodes lamina-associated polypeptide 1 (LAP1), have collectively been implicated in severe cases of cardiomyopathy, muscular dystrophy, dystonia and brain atrophy [9,10,11,12,13,14,15]. Surprisingly, despite increasing evidence pointing to a strong association between LAP1 dysfunction and the development of highly pathological phenotypes, the physiological properties of this NE protein remain poorly understood and, hence, this topic deserves further investigation.

Mammalian LAP1 has been classified as an INM-resident type II integral membrane protein that is structurally constituted by a long N-terminal domain extending into the nucleoplasm, a single transmembrane segment and a short C-terminal domain located in the PNS [16,17,18]. In humans, two *TOR1AIP1* transcripts have been identified, being designated LAP1B [18] and LAP1C, the latter of which results from a downstream alternative transcription initiation site. The corresponding proteins were predicted to differ in the initial portion of their nucleoplasmic domain, which is putatively truncated in LAP1C [19]. A second LAP1B variant lacking an alanine residue at amino acid position 185 (LAP1B ΔA185) has also been described [20,21] and it appears to be functionally similar to full-length LAP1B [22]. Regarding its expression, human LAP1 is found in many cell types, but the relative abundance of LAP1B and LAP1C isoforms varies among different tissues and seems to be developmentally regulated [19].

Over the past few decades, the identification of molecular interactions with diverse proteins has provided some clues about the potential biological roles of mammalian LAP1. For instance, LAP1 has been implicated in the preservation of skeletal myocyte structure by positioning emerin:lamin A complexes at the INM (LAP1:emerin interaction) [23], in the activation of torsin ATPase activity at the NE (LAP1:torsinA interaction) [24,25,26] and in the maintenance of NE structural integrity in interphase cells by anchoring the nuclear lamina to the INM (LAP1:lamins A/C and B1 interaction) [27,28]. Taken together, these findings suggest that, at least in some cases, LAP1 may exert a regulatory action on its binding partners, affecting their localization and/or function in the nucleus. Accordingly, investigating the association of LAP1 with other proteins appears to be a valuable strategy towards uncovering its physiological properties.

Recently, in the laboratory, an in silico study characterized the LAP1 interactome and a total of 38 mammalian protein interactors were described. A gene ontology (GO) term enrichment analysis of the biological processes associated to these LAP1 interactions revealed the process of “regulation of response to DNA damage stimulus” as the most significant one retrieved [29]. In line with this, several proteins included in the LAP1 interaction network had previously been reported to play a role in the DNA damage response (DDR), a tightly coordinated cellular mechanism that detects DNA lesions and induces their repair to ensure genome integrity [30]. Among such LAP1 interactors were, for example, ataxia–telangiectasia mutated protein (ATM), telomeric repeat-binding factor 2 (TRF2), repressor/activator protein 1 (RAP1; also known as TRF2-interacting protein, TRF2IP) and RAP1-interacting factor 1 (RIF1) [29]. ATM is a protein kinase that is at the center of the DDR by phosphorylating multiple substrates involved in cell cycle control, DNA repair, gene regulation or apoptosis in the presence of DNA double-stranded breaks (DSBs) [31]. In turn, the remaining three proteins are components of the mammalian shelterin complex, a specialized nucleoprotein protective structure that promotes telomere capping and maintenance [32].

Of the proteins mentioned above, TRF2 appears to be an attractive target to unravel this novel putative regulatory function of LAP1 in the DDR, given the wide variety of activities that it performs in this biological context. Briefly, as a shelterin complex subunit, TRF2 binds to double-stranded telomeric DNA and facilitates its folding into a t-loop configuration that sequesters the terminal single strand of chromosome ends within the duplex region, thus preventing their misrecognition as DSBs by the DNA damage surveillance system [33,34]. This protective effect on telomere structure is further supported by the ability of TRF2 to physically interact with ATM and repress its activation, which impedes downstream ATM-dependent DDR signaling at the telomeric DNA [35,36,37]. In addition, TRF2 can act cooperatively with its binding partner RAP1 to block the recruitment of DNA repair factors and consequent inappropriate processing of chromosome ends, avoiding the generation of telomere fusions by non-homologous end-joining (NHEJ) [37,38], as well as telomere deletions and the formation of telomere-free chromosome fusions by homologous recombination (HR) [39]. In sharp contrast with this inhibitory action on DDR mechanisms, several lines of evidence indicate that TRF2 is additionally directly involved in DSB repair events occurring in extra-telomeric regions. In short, TRF2 is rapidly and transiently recruited to DNA damage sites located outside of telomeres at a very initial stage of genomic DNA break recognition [40,41,42,43,44]. The efficiency of TRF2 clustering at non-telomeric DNA increases in response to high doses of DSBs with complex lesions (e.g., crosslinking and base damage) [43], suggesting that TRF2 may act as an early sensor of this type of DNA damage and/or as a mediator of its effective resolution. Once at the injured chromatin regions, TRF2 is essential for directing the processing of genomic DSBs towards a more accurate repair pathway of HR, likely through its ability to stimulate intra-chromatid strand invasion [44,45], though a possible role in the fast, error-prone repair pathway of NHEJ has also been suggested [46]. Importantly, the only previous report of an association between LAP1 and TRF2 (and with RAP1 as well) came from a genome-wide study dedicated to the identification of candidate human proteins that bind to the six core shelterin complex subunits [47]. In fact, a knowledge gap exists regarding the physiological significance of LAP1:TRF2 interaction and, given the hypothesized new LAP1 function that emerged from the characterization of its interactome [29], it seems logical to focus on the role of this protein complex in a DDR activation context.

Another aspect worth considering is the nature of the interaction of LAP1 with ATM. Protein phosphorylation is a reversible post-translational modification that plays a major regulatory role in DDR signaling events. It is primarily mediated by ATM, ATM- and Rad3-related protein (ATR) and DNA-dependent protein kinase catalytic subunit (DNA-PKcs), a group of serine/threonine-specific protein kinases that belong to the phosphatidylinositol 3-kinase-related kinase (PIKK) family. ATM and ATR share substrate specificity, as both phosphorylate their downstream target proteins on serines or threonines that are followed by glutamines (i.e., Ser–Gln (SQ) or Thr–Gln (TQ) motifs, respectively) [30]. Notably, several serine residues of human LAP1 located in these consensus sites recognized by ATM and ATR are phosphorylated upon induction of DNA damage, including Ser164, Ser169, Ser255 and Ser267 [48] (numbering relative to LAP1B’s NCBI reference sequence: NP_001254507.1 [49]). This is also true for human TRF2, whose Thr230, Ser410 and Ser422 residues (numbering relative to TRF2′s NCBI reference sequence: NP_005643.2 [50]) are subjected to phosphorylation by ATM and/or ATR under similar biological conditions [41,48]. Interestingly, it has been shown that the Thr230-phosphorylated form of TRF2 specifically accumulates at DSB sites, which suggests that this post-translational modification may be required for its non-telomeric function, possibly by regulating its interaction with other mediators of the DNA repair machinery [41]. Overall, these findings raise the possibility that LAP1:TRF2 complex formation may be modulated by reversible protein phosphorylation when cells react against DNA damage, which deserves further investigation.

Therefore, the main purpose of the work here presented was to clarify the physiological properties of human LAP1 by studying the functional relevance of its interaction with TRF2 in a biological context of DDR activation, using the HeLa cell line as a primary model system. In this study, we validated the binding of LAP1 to TRF2 both in vitro and in cells exposed to DNA damage-inducing treatments. Moreover, it was possible to show that this interaction is regulated by protein phosphorylation, occurring preferentially when LAP1 is in a phosphorylated state. Additionally, it was demonstrated that the nuclear protein levels of both LAP1 and TRF2 increase in response to genotoxic insults and that they co-localize with each other as well as with histone variant H2AX phosphorylated at Ser139 (γ-H2AX) and ATM phosphorylated at Ser1981 (pATM^S1981^)—two important DDR factors—not only at the NE but also in intranuclear regions. Finally, we reported the accumulation of LAP1 in the periphery of nuclear blebs and micronuclei, where it co-exists with TRF2 and damaged chromatin-associated proteins.

## 2. Materials and Methods

### 2.1. Antibodies

Several primary antibodies were used to detect human target proteins by immunoblotting (IB), immunocytochemistry (ICC) and/or co-immunoprecipitation (co-IP), namely: mouse monoclonal anti-γ-H2AX (Millipore (05-636), Darmstadt, Germany; 1:500 for IB and ICC); mouse monoclonal anti-pATM^S1981^ (Santa Cruz Biotechnology (sc-47739), Heidelberg, Germany; 1:200 for ICC); rabbit polyclonal anti-LAP1 (Goodchild and Dauer [51]; 1:20000 for IB; 0.2 µL per 2000 µg of protein for co-IP); rabbit polyclonal anti-LAP1 (Atlas Antibodies (HPA050546), Bromma, Sweden; 1:150 for ICC); mouse monoclonal anti-TRF2 (Abcam (ab13579), Cambridge, United Kingdom (UK); 1:2000 for IB; 1:500 for ICC; 7 µL per 2000 µg of protein for co-IP); goat polyclonal anti-TRF2 (Abcam (ab13589); 1:200 for ICC); mouse monoclonal anti-RAP1 (Abcam (ab14404); 1:5000 for IB; 5 µL per 2000 µg of protein for co-IP); and mouse monoclonal anti-β-tubulin (Invitrogen (32-2600), Thermo Fisher Scientific, Waltham, Massachusetts, United States of America (USA); 1:1000 for IB). Additionally, depending on the technique employed, specific secondary antibodies were used to recognize primary antibodies. For immunoblotting, horseradish peroxidase (HRP)-linked horse anti-mouse immunoglobulin G (IgG) (Cell Signaling Technology (7076), Leiden, The Netherlands; 1:10000), HRP-linked goat anti-rabbit IgG (Cell Signaling Technology (7074); 1:10000), HRP-linked rat anti-mouse TrueBlot IgG (Rockland (18-8817-30), Limerick, Pennsylvania, USA; 1:1000) and HRP-linked mouse anti-rabbit TrueBlot IgG (Rockland (18-8816-33); 1:1000) secondary antibodies were utilized for enhanced chemiluminescence (ECL) detection. For immunocytochemistry, the following secondary antibodies were used: Alexa Fluor 488-conjugated goat anti-mouse IgG (Invitrogen (A-11001); 1:300), Alexa Fluor 594-conjugated goat anti-mouse IgG (Invitrogen (A-11005); 1:300), Alexa Fluor 488-conjugated goat anti-rabbit IgG (Invitrogen (A-11008); 1:300) and Alexa Fluor 594-conjugated donkey anti-goat IgG (Invitrogen (A-11058); 1:200).

### 2.2. Cell Culture Procedures

The human HeLa cell line (American Type Culture Collection (ATCC) CCL-2, Manassas, Virginia, USA) was cultured in Dulbecco’s modified Eagle medium (DMEM; Gibco, Thermo Fisher Scientific, Waltham, Massachusetts, USA) supplemented with 10% inactivated fetal bovine serum (FBS; Gibco) and 1% antibiotic/anti-mycotic (Gibco). The human SH-SY5Y cell line (ATCC CRL-2266) was grown in minimum essential medium (MEM; Gibco)/Ham’s F-12 nutrient mixture (Gibco) supplemented with 10% FBS, 1% sodium pyruvate (Sigma-Aldrich, Saint Louis, Missouri, USA) and 1% antibiotic/anti-mycotic. HeLa and SH-SY5Y cells were maintained at 37 °C in a humidified atmosphere with 5% CO_2_ and subcultured when a confluency of 80%–90% was reached.

#### Cell Treatments

For the induction of genome-wide DNA damage, HeLa cells were exposed to hydrogen peroxide (H_2_O_2_; Acros Organics, Geel, Belgium) or bleomycin (Santa Cruz Biotechnology, Heidelberg, Germany). Prior to the assays (i.e., immunoblotting, co-immunoprecipitation and immunocytochemistry), cells were seeded and cultured in standard conditions for 16 h before exposure to the DNA-damaging agents. After that period, cells were incubated at 37 °C in fresh culture medium containing different concentrations of H_2_O_2_ or bleomycin over several time points. A control condition was used in each experiment, in which no H_2_O_2_ or bleomycin was added to the culture medium. In turn, for the inhibition of protein phosphatase activity, HeLa and SH-SY5Y cells were treated with okadaic acid (Calbiochem, Merck, Darmstadt, Germany). Similarly, before the assays (i.e., co-immunoprecipitation), cells were plated and grown for 16 h, after which they were either exposed to the phosphatase inhibitor or incubated in normal culture medium at 37 °C.

- H_2_O_2_ Treatment

For immunoblotting, HeLa cells were seeded at a density of 1.25 × 10^5^ cells in 12-well plates and posteriorly treated with 50, 100, 150 or 250 µM of H_2_O_2_ for 6 h or 12 h. For co-immunoprecipitation, cells were standardly cultured in 100 mm dishes until a confluency of 90% was reached and subsequently exposed to 150 µM of H_2_O_2_ for 12 h.

- Bleomycin Treatment

In one set of experiments, the exposure of HeLa cells to bleomycin was performed through long time periods and was not followed by a recovery phase (hereafter referred to as long-term bleomycin treatment). For immunoblotting, 7.5 × 10^4^ cells were initially grown in 12-well plates and later incubated with 2, 10 or 50 µg/mL of bleomycin for 24 h or 48 h. Alternatively, a shorter bleomycin exposure time was used in another set of experiments, being followed by distinct recovery periods (hereafter referred to as short-term bleomycin treatment). For immunoblotting, HeLa cells were plated either at a density of 2 × 10^5^ cells in 12-well plates to collect whole cell lysates or at a density of 5.25 × 10^5^ cells in 6-well plates to obtain nuclear protein extracts. For immunocytochemistry, 4.25 × 10^5^ cells were seeded in 6-well plates containing glass coverslips. During treatment, cells were cultured in the presence of 50 or 200 µg/mL of bleomycin for 30 min and then incubated in fresh culture medium for 45 min, 3 h or 6 h to facilitate their recovery from inflicted DNA damage. For co-immunoprecipitation, cells were standardly grown in 100 mm dishes until a confluency of 90% was reached and posteriorly treated with 200 µg/mL of bleomycin for 30 min, followed by 6 h of recovery in normal culture medium.

- Okadaic Acid Treatment

For co-immunoprecipitation, HeLa and SH-SY5Y cells were grown in standard conditions in 100 mm dishes until a confluency of 90% was reached, after which they were exposed to 500 nM of okadaic acid for 3 h.

### 2.3. Preparation of Whole Cell Lysates

Following H_2_O_2_ and bleomycin treatments, HeLa cells were lysed by resuspension in boiling 1% sodium dodecyl sulfate (SDS). Whole cell lysates were boiled at 90 °C for 10 min and sonicated on ice for 10 s (0.5 cycles, 60% amplitude). Total protein concentration was determined using the Pierce bicinchoninic acid (BCA) protein assay kit (Thermo Scientific, Thermo Fisher Scientific, Waltham, Massachusetts, USA), according to the manufacturer’s instructions. Afterwards, whole cell lysates were separated by sodium dodecyl sulfate–polyacrylamide gel electrophoresis (SDS–PAGE) and analyzed by immunoblotting (see Section 2.7).

### 2.4. Preparation of Nuclear Lysates by Subcellular Fractionation

The subcellular fractionation method previously described by Suzuki et al. [52] was performed. For the blot overlay assay, the pellet corresponding to the nuclear protein fraction was resuspended in ice-cold 1× phosphate buffered saline (PBS) and sonicated on ice for 5 s (0.5 cycles, 60% amplitude). This nuclear extract from HeLa cells was posteriorly incubated with a nitrocellulose membrane containing samples enriched for human LAP1B proteins (see Section 2.5). Alternatively, in the experiments wherein HeLa cells were subjected to the short-term bleomycin treatment, the nuclear pellet was resuspended in boiling 1% SDS, boiled at 90 °C for 10 min and sonicated on ice for 10 s (0.5 cycles, 60% amplitude) before SDS–PAGE and immunoblotting analysis (see Section 2.7).

### 2.5. Blot Overlay

Human full-length LAP1B and the shorter variant LAP1B ∆A185 (generated by site-directed mutagenesis) were synthesized by in vitro transcription/translation (IVT) using the TnT-coupled transcription/translation system (Promega, Madison, WI, USA), as previously reported by Santos et al. [22]. LAP1B–IVT and LAP1B ∆A185–IVT samples were resolved by 10% SDS–PAGE and electrotransferred onto a nitrocellulose membrane (0.2 µm pore size; GE Healthcare, Buckinghamshire, UK). For the overlay assay, the membrane was blocked in 3% bovine serum albumin (BSA)/1× Tris-buffered saline with 0.1% Tween-20 (TBS-T) for 1 h, after which it was overlaid with HeLa cells’ nuclear extract for 1 h. Next, the membrane was incubated with a TRF2-specific primary antibody (see Section 2.1) in 3% BSA/1× TBS-T for 2 h, followed by incubation with an HRP-conjugated secondary antibody (see Section 2.1) in 5% fat-free dry milk/1× TBS-T for 1 h. The protein bands corresponding to LAP1B and LAP1B ∆A185 were visualized in a ChemiDoc Imaging System (Bio-Rad, Hercules, CA, USA) by ECL detection.

### 2.6. Co-Immunoprecipitation

Co-immunoprecipitation assays were performed under different conditions, namely in a baseline state, upon DNA damage induction through H_2_O_2_ or bleomycin exposure and after protein phosphatase activity inhibition via okadaic acid treatment. HeLa and SH-SY5Y cells were collected in ice-cold 3-((3-cholamidopropyl) dimethylammonio)-1-propanesulfonate (CHAPS) lysis buffer (50 mM Tris-HCl, pH = 8; 120 mM NaCl; 4% CHAPS) supplemented with 1% protease inhibitor cocktail (Sigma-Aldrich, Saint Louis, Missouri, USA). Cell lysates were sonicated on ice for 5 s (0.5 cycles, 60% amplitude) and the total protein concentration measured using the Pierce BCA protein assay kit. Before their use, Dynabeads Protein G (Invitrogen, Thermo Fisher Scientific, Waltham, Massachusetts, USA) were washed with ice-cold 3% BSA/1× PBS. Specific primary antibodies against LAP1, TRF2 and RAP1 (see Section 2.1), as well as control rabbit and mouse IgGs (Invitrogen), were cross-linked to Dynabeads Protein G for 2 h at 4 °C. A cell lysate sample containing 2000 µg of protein was pre-cleared with Dynabeads Protein G for 1 h at 4 °C and then incubated with Dynabeads Protein G–antibody (or control IgG) conjugates overnight at 4 °C. The resultant immunoprecipitates were eluted from Dynabeads Protein G by boiling in 1× loading buffer (62.5 mM Tris, pH = 6.8; 2% SDS; 10% glycerol; 5% β-mercaptoethanol; 0.0025% bromophenol blue) at 90 °C for 10 min, being subsequently resolved by SDS–PAGE and analyzed by immunoblotting (see Section 2.7) or high-performance liquid chromatography–mass spectrometry (HPLC–MS) (see Section 2.8).

### 2.7. SDS–PAGE and Immunoblotting

Whole cell lysates and nuclear lysates were separated on a gradient (5%–20%) polyacrylamide gel and immunoprecipitated proteins on a 10% polyacrylamide gel. Prior to loading, samples were boiled in 1× loading buffer at 90 °C for 10 min. After SDS–PAGE, proteins were electrophoretically transferred onto nitrocellulose membranes. For immunoblotting, upon blocking in 5% BSA/1× TBS-T for 3 h, membranes were incubated with specific primary antibodies against γ-H2AX, LAP1, TRF2, RAP1 and β-tubulin (see Section 2.1) in 3% BSA/1× TBS-T for 2 h, followed by overnight incubation at 4 °C. On the next day, membranes were incubated with HRP-conjugated secondary antibodies (see Section 2.1) in 5% fat-free dry milk/1× TBS-T for 1 h. To detect protein bands by ECL, immunoblots were scanned in a ChemiDoc Imaging System. Additionally, membranes were stained with Ponceau S solution (5% acetic acid; 0.1% Ponceau S) to determine the total protein content in each sample [53]. Quantification of γ-H2AX, LAP1 and TRF2 protein levels was achieved with the Image Lab software (Bio-Rad, Hercules, California, USA) using Ponceau S staining or β-tubulin expression as protein loading control for data normalization. Relative protein levels were calculated by comparing the different treatment conditions with their respective control.

### 2.8. HPLC–MS

SDS–PAGE gels containing immunoprecipitated protein samples from okadaic acid-treated SH-SY5Y cells were stained with Coomassie blue and the two protein bands comprising LAP1B (≈68 kDa) and LAP1C (≈56 kDa) were excised from the gels. Following destaining, the protein bands were tryptically digested and the resultant peptides analyzed in a Ultimate 3000 nano-HPLC system (Dionex, Thermo Fisher Scientific, Waltham, Massachusetts, USA) coupled to a Q Exactive quadrupole-orbitrap mass spectrometer (Thermo Fisher Scientific), as previously described by Santos et al. [19].

### 2.9. Immunocytochemistry

HeLa cells subjected to the short-term bleomycin treatment were fixed using 3.7% paraformaldehyde (PFA) for 20 min and posteriorly permeabilized with 0.2% Triton X-100/1× PBS for 10 min. After blocking in 3% BSA/1× PBS for 2 h, cells were incubated with specific primary antibodies against γ-H2AX, pATM^S1981^, LAP1 and TRF2 (see Section 2.1) in 3% BSA/1× PBS for 1–2 h, followed by incubation with Alexa Fluor 488- and Alexa Fluor 594-conjugated secondary antibodies (see Section 2.1) in 3% BSA/1× PBS for 1 h. Coverslips were mounted on microscope slides using 4′,6-diamidino-2-phenylindole (DAPI)-containing Vectashield anti-fade mounting medium (Vector Laboratories, Burlingame, California, USA).

### 2.10. Confocal Microphotograph Acquisition and Analysis

Immunocytochemistry preparations were visualized using an LSM 880 confocal laser scanning microscope with Airyscan (Zeiss, Jena, Germany) and a 63×/1.4 oil immersion objective. Fluorescence excitation was achieved with the 405 nm (DAPI), 488 nm (Alexa Fluor 488) and 561 nm (Alexa Fluor 594) laser lines. Microphotographs were acquired in multiple optical sections in the Z-axis to produce z-stacks. For quantifying the corrected total cell fluorescence (CTCF) levels of γ-H2AX, LAP1 and TRF2 in HeLa cells’ nuclei, the ImageJ software [54] was employed. In brief, after creating a Z projection using the sum intensity method, nuclei were detected as regions of interest (ROIs) by applying a threshold to the DAPI channel. CTCF levels were calculated for each segmented nucleus by subtracting the corresponding fluorescence of adjacent background from the integrated density in both the green and red channels. To obtain relative CTCF levels, the treatment conditions were accordingly compared to control. In turn, for evaluating LAP1/γ-H2AX, TRF2/γ-H2AX and LAP1/TRF2 co-localization, a pixel intensity spatial correlation analysis was carried out using the JACoP plugin [55] of ImageJ. In short, each segmented nucleus in a z-stack was subjected to Costes’ statistical significance test [56] to check for true co-localization among the green and red fluorescent dyes, excluding cases of random color overlap. Nuclei with a *p*-value > 0.95 were considered to have statistically significant co-localization and further analyzed individually. The green and red channels were split and their threshold adjusted to identify labeled nuclear compartments. Next, the Pearson’s correlation coefficient (PCC) was measured, providing information regarding the degree to which the signal intensities of two immunostained proteins were linearly related to each other. The extent of co-occurrence, representing the fraction of one immunostained protein spatially overlapped with another protein, was also determined using Manders’ co-localization coefficients M_1_ (proportion of red fluorophore overlapping with green fluorophore) and M_2_ (proportion of green fluorophore overlapping with red fluorophore) [57].

### 2.11. Statistical Analysis

For immunoblotting and immunocytochemistry quantitative results, data were expressed as mean ± standard error of the mean (SEM) of, at least, three independent experiments. All statistical analyses were performed using the GraphPad Prism 7 software (GraphPad Software, San Diego, California, USA). For the comparison of protein levels between control and several treatment groups, the non-parametric Kruskal–Wallis test was applied, followed by the Dunn’s multiple comparison test. For the comparison of CTCF levels as well as PCC, M_1_ and M_2_ values between control and one treatment group, the non-parametric Mann–Whitney *U* test was employed. Values of *p* < 0.05 were considered statistically significant.

## 3. Results

### 3.1. LAP1 and TRF2 Interact In Vitro

**Figure 1:**
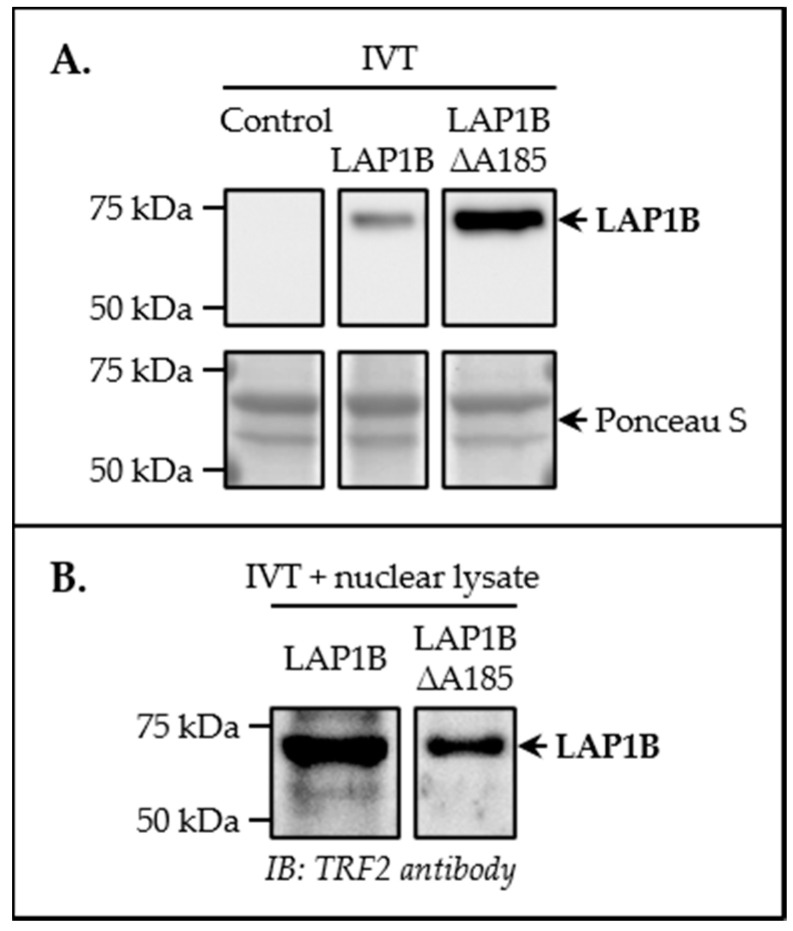
Validation of in vitro LAP1:TRF2 complex formation. (**A**) Successful in vitro translation of LAP1B variants. The membrane comprising IVT-derived samples of a negative control (i.e., empty vector) (left), full-length LAP1B (middle) and the shorter variant LAP1B ∆A185 (right) were incubated with a LAP1-specific antibody to confirm the presence of LAP1B in both LAP1B–IVT and LAP1B ∆A185–IVT samples. Ponceau S staining was used as protein loading control. (**B**) Blot overlay assay. The membrane containing LAP1B–IVT (left) and LAP1B ∆A185–IVT (right) samples was overlaid with HeLa cells’ nuclear lysate, followed by TRF2 immunoblotting analysis. A specific antibody against TRF2 detected a protein band of ≈ 68 kDa in each sample, corresponding to the molecular weight of both LAP1B variants. IB, immunoblotting; IVT, in vitro transcription/translation; LAP1, lamina-associated polypeptide 1; TRF2, telomeric repeat-binding factor 2.

In a previous large-scale protein–protein interaction screen searching for candidate telomere regulatory proteins, a putative association between the shelterin complex subunit TRF2 and the INM protein LAP1 emerged for the first time [47]. This prompted us to intensify the study of this proposed novel protein complex with the intent of elucidating the physiological properties of human LAP1. Firstly, an in vitro blot overlay assay to validate TRF2 as a LAP1-binding partner was performed. Briefly, two samples of human full-length LAP1B (variant 1) and LAP1B ∆A185 (variant 2) synthesized by IVT (Figure 1A) were separated by SDS–PAGE and electrotransferred onto a nitrocellulose membrane. HeLa cells’ whole lysates were subjected to subcellular fractionation so as to isolate the nuclear protein fraction, permitting to obtain a cellular extract highly enriched for TRF2 (data not shown), among other nuclear proteins, that was subsequently overlaid on the blot containing LAP1B–IVT and LAP1B ∆A185–IVT. For each sample, a band of approximately 68 kDa, which corresponds to the molecular weight of these LAP1 proteins, was detected by immunoblotting using a TRF2-specific antibody (Figure 1B). These results indicate that TRF2, a protein present in the nuclear lysate, was able to bind in vitro to both LAP1B variants immobilized on the blot’s surface. Therefore, we infer that this interaction involves a direct physical association between human TRF2 and LAP1.

### 3.2. LAP1:TRF2 Interaction Occurs in Response to DNA Damage and Is Regulated by LAP1 Phosphorylation In Vivo

**Figure 2:**
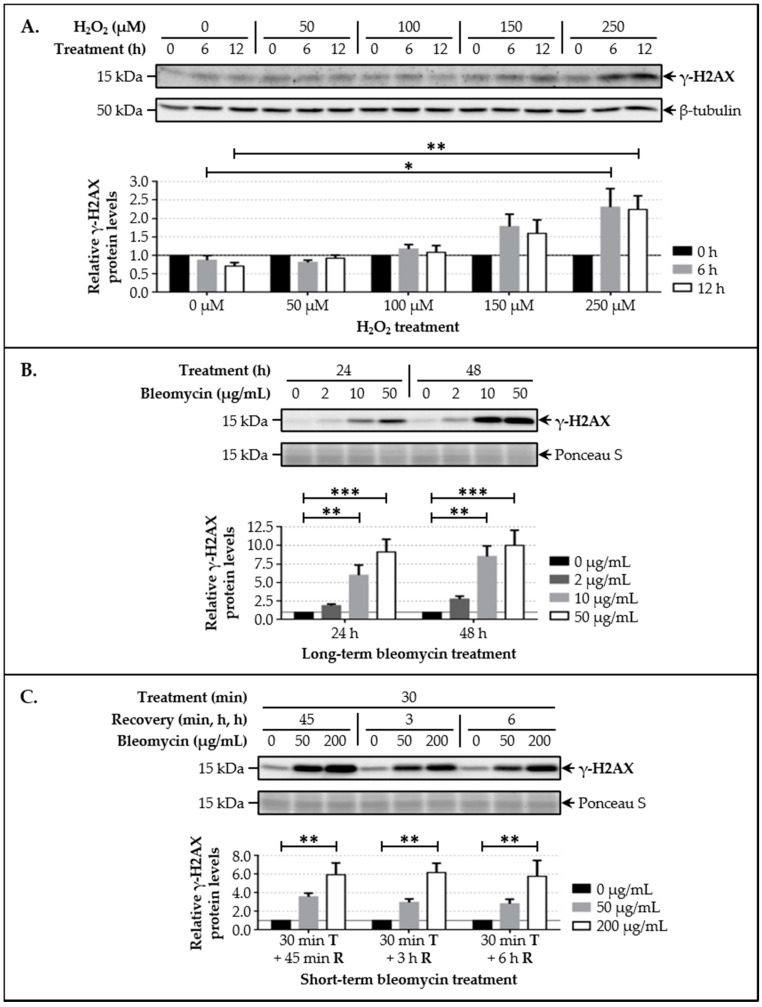
Detection of cellular DNA damage induced by H_2_O_2_ and bleomycin treatments in the human HeLa cell line. (**A**) Relative γ-H2AX protein levels after cell exposure to 50, 100, 150 and 250 µM of H_2_O_2_ for 6 h and 12 h (estimated in relation to untreated cells in the beginning of the experiment (0 h)). (**B**) Relative γ-H2AX protein levels after cell exposure to 2, 10 and 50 µg/mL of bleomycin for 24 h and 48 h (estimated in relation to untreated cells (0 µg/mL) at each timepoint). (**C**) Relative γ-H2AX protein levels after cell exposure to 50 and 200 µg/mL of bleomycin for 30 min (T), followed by 45 min, 3 h and 6 h of recovery (R) (estimated in relation to untreated cells (0 µg/mL) at each timepoint). In all treatments, HeLa cells’ whole lysates were analyzed by immunoblotting using a specific antibody against γ-H2AX to measure DNA damage levels. A representative blot is shown for each treatment. Quantitative data are presented as mean ± SEM (*n* = 3 (**A**,**B**) or *n* = 4 (**C**)). Before determining relative γ-H2AX protein levels, data normalization was performed using β-tubulin immunoblotting (**A**) or Ponceau S staining (**B**,**C**) as protein loading control. * *p* < 0.05, ** *p* < 0.01 and *** *p* < 0.001 for comparisons between control and treatment conditions using the Kruskal–Wallis test followed by the Dunn’s multiple comparison test. γ-H2AX, histone variant H2AX phosphorylated at Ser139; H_2_O_2_, hydrogen peroxide; SEM, standard error of the mean.

After showing that a protein complex formed by LAP1 and TRF2 occurs *in vitro*, we investigated the establishment of this interaction in human cells. We hypothesized that it could be constitutively active in the cell or, instead, be triggered by a specific signaling event, more precisely by DDR activation, given the previously reported inhibitory [33,34,35,36,37,38,39] and stimulatory [40,41,42,43,44,45,46] roles of TRF2 as well as the recently suggested involvement of LAP1 [29] in this biological process. Before directly addressing this question, cellular models of DNA damage induction were developed; these consisted in exposing HeLa cells to different concentrations of H_2_O_2_—a reactive oxygen species (ROS)—or bleomycin—a radiomimetic anticancer drug—for distinct time periods. Regarding the H_2_O_2_ treatment, cells were cultured in the presence of 50, 100, 150 or 250 µM of H_2_O_2_ for 6 h or 12 h. In the case of bleomycin, two kinds of treatment were performed: (i) a long-term exposure to 2, 10 or 50 µg/mL of bleomycin that lasted 24 h or 48 h; and (ii) a shorter incubation with 50 or 200 µg/mL of bleomycin over 30 min, followed by a recovery phase of 45 min, 3 h or 6 h. Importantly, the histone variant H2AX is one of the earliest substrates phosphorylated (on Ser139) by PIKK family protein kinases in response to DNA breaks, yielding γ-H2AX, which promotes the recruitment of additional DDR factors to damaged chromatin sites; as such, the nuclear accumulation of this modified form of H2AX is widely recognized as a DNA damage marker [58]. Accordingly, we evaluated γ-H2AX protein levels in HeLa cells’ whole lysates by immunoblotting to monitor the degree of DNA damage in each experimental condition. The results revealed that cell exposure to 250 µM and, to a lesser extent, 150 µM of H_2_O_2_ caused an increase in γ-H2AX levels at both 6 h and 12 h timepoints relative to the control group, being statistically significant with the highest concentration used (Figure 2A). Moreover, we found significantly increased γ-H2AX levels in cells incubated with 10 and 50 µg/mL of bleomycin over 24 h and 48 h (Figure 2B), as well as in those cultured with 200 µg/mL of bleomycin for 30 min (regardless of the recovery phase’s duration) (Figure 2C), in comparison with untreated cells. Overall, it is feasible to conclude that the three treatments tested have DNA damage-inducing effects on HeLa cells in a dose-dependent manner.

**Figure 3:**
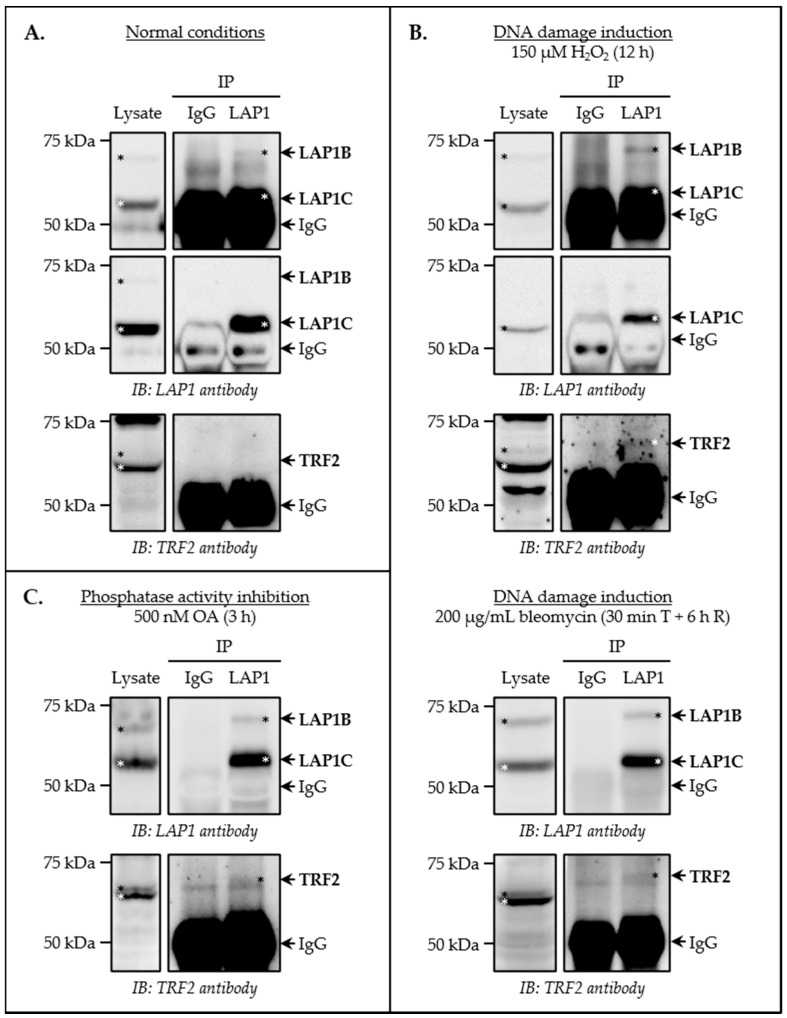
Validation of in vivo LAP1:TRF2 complex formation upon induction of DNA damage and its regulation by protein phosphorylation in the human HeLa cell line. (**A**) LAP1 co-immunoprecipitation assay in control conditions. (**B**) LAP1 co-immunoprecipitation assay after cell exposure to H_2_O_2_ (150 µM for 12 h) and bleomycin (200 µg/mL for 30 min (T), followed by 6 h of recovery (R)), two DNA-damaging agents. (**C**) LAP1 co-immunoprecipitation assay after cell exposure to okadaic acid (500 nM for 3 h), a serine/threonine-specific protein phosphatase inhibitor. In all assays, HeLa cells’ whole lysates were immunoprecipitated using a Dynabeads Protein G-conjugated LAP1-specific antibody. The corresponding negative controls were performed by incubating total protein extracts with rabbit IgG bound to Dynabeads Protein G. The presence of LAP1 (upper blots in panels A and B (H_2_O_2_): less exposure time; lower blots in panels A and B (H_2_O_2_): more exposure time) and TRF2 in the immunoprecipitates (right), as well as in the initial whole cell lysates (left), was analyzed by immunoblotting using specific antibodies. Asterisks indicate visible LAP1B (≈68 kDa), LAP1C (≈56 kDa) and TRF2 (≈69/65 kDa) protein bands in each blot. H_2_O_2_, hydrogen peroxide; IB, immunoblotting; IgG, immunoglobulin G; IP, immunoprecipitation; LAP1, lamina-associated polypeptide 1; OA, okadaic acid; TRF2, telomeric repeat-binding factor 2. Validation of in vivo LAP1:TRF2 complex formation upon induction of DNA damage and its regulation by protein phosphorylation in the human HeLa cell line. (**A**) LAP1 co-immunoprecipitation assay in control conditions. (**B**) LAP1 co-immunoprecipitation assay after cell exposure to H_2_O_2_ (150 µM for 12 h) and bleomycin (200 µg/mL for 30 min (T), followed by 6 h of recovery (R)), two DNA-damaging agents. (**C**) LAP1 co-immunoprecipitation assay after cell exposure to okadaic acid (500 nM for 3 h), a serine/threonine-specific protein phosphatase inhibitor. In all assays, HeLa cells’ whole lysates were immunoprecipitated using a Dynabeads Protein G-conjugated LAP1-specific antibody. The corresponding negative controls were performed by incubating total protein extracts with rabbit IgG bound to Dynabeads Protein G. The presence of LAP1 (upper blots in panels A and B (H_2_O_2_): less exposure time; lower blots in panels A and B (H_2_O_2_): more exposure time) and TRF2 in the immunoprecipitates (right), as well as in the initial whole cell lysates (left), was analyzed by immunoblotting using specific antibodies. Asterisks indicate visible LAP1B (≈68 kDa), LAP1C (≈56 kDa) and TRF2 (≈69/65 kDa) protein bands in each blot. H_2_O_2_, hydrogen peroxide; IB, immunoblotting; IgG, immunoglobulin G; IP, immunoprecipitation; LAP1, lamina-associated polypeptide 1; OA, okadaic acid; TRF2, telomeric repeat-binding factor 2.

Once different human cellular models of the DDR were established, a series of co-immunoprecipitation assays were carried out to study the in vivo occurrence of the LAP1:TRF2 complex. First, we investigated if these proteins interact constitutively in a physiological context. In brief, whole cell lysates collected from HeLa cells in a baseline state were immunoprecipitated with a specific antibody against LAP1 and the presence of TRF2 in the immunoprecipitates was assessed by immunoblotting. As a control, total protein extracts were incubated with rabbit IgG. Our results revealed that, even though LAP1B and LAP1C were efficiently immunoprecipitated, TRF2 was not detected among the proteins that bind to these LAP1 isoforms under control conditions (Figure 3A). To rule out the possibility that this absence could be related with the human cell line used, a similar assay was conducted in SH-SY5Y cells. Again, we were not successful at co-immunoprecipitating TRF2 along with LAP1 in normal conditions (Supplementary Figure S1A). Additionally, in both experiments, RAP1 immunoblotting was performed to search for a putative interaction between this shelterin complex subunit and LAP1, which had previously been suggested in a genome-wide telomere interactome study [47]. However, the presence of RAP1 in LAP1-associated immunoprecipitates was not observed, neither in SH-SY5Y cells (Supplementary Figure S1A) nor in HeLa cells (Supplementary Figure S2A). To further confirm that our findings were not related with the experimental design of the assay and/or its technical execution, SH-SY5Y cells’ whole lysates were immunoprecipitated with TRF2- and RAP1-specific antibodies. In both cases, we demonstrated the well-known constitutive TRF2:RAP1 association in baseline conditions, but not an interaction between either one of these telomeric proteins and LAP1 (Supplementary Figure S1B,C). Considering all the above results, we postulated that LAP1:TRF2 complex formation probably has a transient nature and may require a specific stimulus to trigger its occurrence in human cells.

To test our hypothesis that the binding of LAP1 to TRF2 is stimulated by activation of the DDR signaling cascade, LAP1 co-immunoprecipitation assays in HeLa cells’ whole lysates collected after exposure to H_2_O_2_ or bleomycin were performed. The following DNA-damaging treatment conditions were used: (i) 150 µM of H_2_O_2_ for 12 h; and (ii) 200 µg/mL of bleomycin for 30 min, followed by 6 h of recovery. Remarkably, TRF2 was efficiently co-immunoprecipitated with LAP1 upon DNA damage induction mediated either by H_2_O_2_ (Figure 3B, top panel) or by bleomycin (Figure 3B, bottom panel). On the other hand, RAP1 was not detected amongst the LAP1-interacting proteins present in the immunoprecipitates, as evaluated in H_2_O_2_ experimental conditions (Supplementary Figure S2B). Accordingly, these results confirm that the LAP1:TRF2 interaction is formed in vivo and, more importantly, show that it is established when the biological DDR process occurs.

Following the in vitro and in vivo validation of the protein complex comprising LAP1 and TRF2, we endeavored to decipher the molecular mechanisms governing its formation in human cells. Based on prior studies that discovered several ATM/ATR-recognized phosphorylation sites in LAP1 [48] and TRF2 [41,48] that are responsive to DNA damage, it is reasonable to deduce that their interaction may be regulated by protein phosphorylation. To explore this issue, a LAP1 co-immunoprecipitation assay was carried out in whole cell lysates from HeLa cells exposed to 500 nM of okadaic acid for 3 h. Under these conditions, okadaic acid inhibits the activity of both serine/threonine-specific protein phosphatases 1 (PP1) and 2A (PP2A) [19,22], culminating in increased levels of hyperphosphorylated forms of their substrates. Interestingly, similar to the results obtained when cells were incubated with DNA-damaging concentrations of H_2_O_2_ or bleomycin (Figure 3B and Supplementary Figure S2B), we detected TRF2 in LAP1-associated immunoprecipitates following exposure to this phosphatase inhibitor (Figure 3C), but, once again, RAP1 was not co-immunoprecipitated with LAP1 (Supplementary Figure S2C). Taking into account that LAP1 dephosphorylation on Ser306 and Ser310 residues has previously been reported to be mediated by PP1 [19] and that, under the conditions specified above, okadaic acid treatment is known to cause an increase in the phosphorylation level of the two human LAP1 isoforms [19,22], our data reveal that LAP1 binds preferentially to TRF2 in vivo when it is phosphorylated.

Subsequently, an HPLC–MS-based approach was used to search for LAP1-interacting proteins in a context of PP1/PP2A phosphatase activity inhibition. Briefly, after LAP1 co-immunoprecipitation in SH-SY5Y cells’ whole lysates collected in a baseline state or upon incubation with okadaic acid (500 nM for 3 h), the protein bands containing LAP1B (≈ 68 kDa) and LAP1C (≈ 56 kDa) separated by SDS–PAGE were analyzed by HPLC–MS. Using this strategy, a total of 22 unique LAP1 peptides (Table 1), which align with the known sequence of human LAP1B throughout its extension (NCBI reference sequence: NP_001254507.1 [49]) (Supplementary Figure S3A), were identified among all the conditions tested. In addition to the detection of LAP1 peptides, this strategy permitted the identification of LAP1-binding proteins whose molecular weight is similar to that of LAP1 isoforms, as are the cases of TRF2 (≈69/65 kDa) and RAP1 (≈56 kDa), among others. We found two unique TRF2 peptides enriched in immunoprecipitated LAP1 samples when PP1 and PP2A were inhibited, but not in those of the control co-immunoprecipitation (Table 1), while the existence of RAP1 peptides was not validated in either condition. A closer analysis of these TRF2 peptides revealed that one aligns with the telomeric repeat-binding factor homology (TRFH) domain and the other with a flexible hinge domain containing a RAP1-binding motif (NCBI reference sequence: NP_005643.2 [50]) (Supplementary Figure S3B). Thus, the presented data unequivocally identify TRF2 as a LAP1 interactor in vivo, more specifically in a cellular context that triggers the phosphorylation of this NE protein.

In summary, the data obtained support two main conclusions, namely that: (i) LAP1 and TRF2 are induced to form a protein complex in human cells during a biological response to DNA damage; and (ii) this interaction is regulated by protein phosphorylation, being favored by an increase in phosphorylated LAP1 levels.

### 3.3. LAP1 and TRF2 Protein Levels Increase Upon Induction of DNA Damage In Vivo

**Figure 4:**
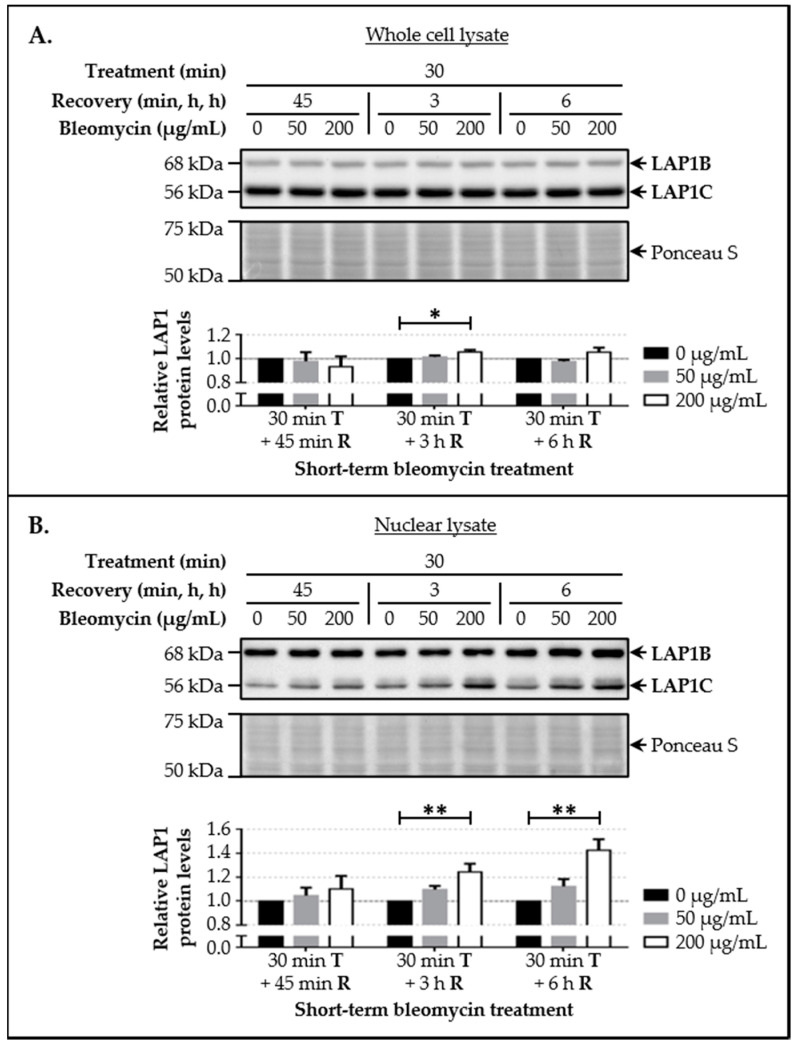
Evaluation of the effects of DNA damage induced by a short-term bleomycin treatment on LAP1 protein levels in the human HeLa cell line. (**A**) Relative LAP1 protein levels in whole cell lysates after cell exposure to 50 and 200 µg/mL of bleomycin for 30 min (T), followed by 45 min, 3 h and 6 h of recovery (R) (estimated in relation to untreated cells (0 µg/mL) at each timepoint). (**B**) Relative LAP1 protein levels in nuclear lysates in the same experimental conditions specified in (**A**). Following treatment, HeLa cells’ total and nuclear extracts were analyzed by immunoblotting using a LAP1-specific antibody to evaluate their protein levels. A representative blot is shown for each type of cellular lysate used. Quantitative data are presented as mean ± SEM (*n* = 4). Before determining relative LAP1 protein levels, data normalization was performed using Ponceau S staining as protein loading control. * *p* < 0.05 and ** *p* < 0.01 for comparisons between control and treatment conditions using the Kruskal–Wallis test followed by the Dunn’s multiple comparison test. LAP1, lamina-associated polypeptide 1; SEM, standard error of the mean.

**Figure 5:**
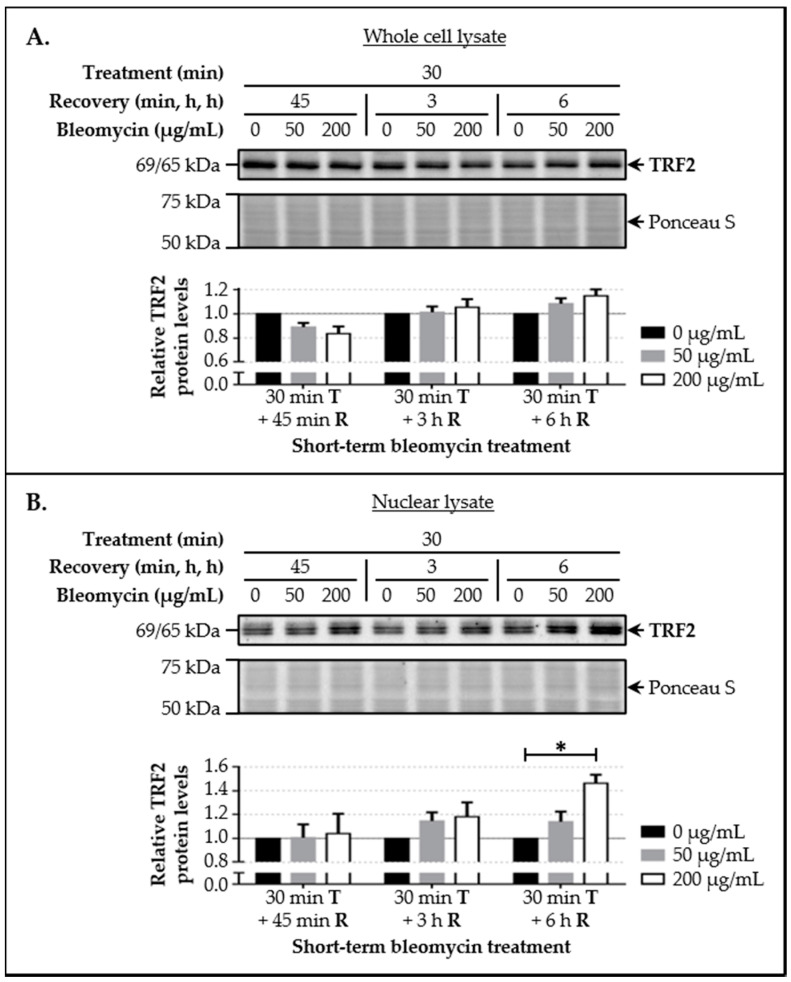
Evaluation of the effects of DNA damage induced by a short-term bleomycin treatment on TRF2 protein levels in the human HeLa cell line. (**A**) Relative TRF2 protein levels in whole cell lysates after cell exposure to 50 and 200 µg/mL of bleomycin for 30 min (T), followed by 45 min, 3 h and 6 h of recovery (R) (estimated in relation to untreated cells (0 µg/mL) at each timepoint). (**B**) Relative TRF2 protein levels in nuclear lysates in the same experimental conditions specified in (**A**). Following treatment, HeLa cells’ total and nuclear extracts were analyzed by immunoblotting using a specific antibody against TRF2 to evaluate their protein levels. A representative blot is shown for each type of cellular lysate used. Quantitative data are presented as mean ± SEM (*n* = 4). Before determining relative TRF2 protein levels, data normalization was performed using Ponceau S staining as protein loading control. * *p* < 0.05 for comparisons between control and treatment conditions using the Kruskal–Wallis test followed by the Dunn’s multiple comparison test. SEM, standard error of the mean; TRF2, telomeric repeat-binding factor 2.

Given the evidence supporting that LAP1 interacts in vivo with TRF2 in the presence of high γ-H2AX levels indicative of DDR activation, the subsequent aim was to evaluate their endogenous protein levels by immunoblotting in HeLa cells following a DNA-damaging treatment. After exposure to different concentrations of bleomycin (50 and 200 µg/mL) for a short time period (30 min), cells were allowed to recover over distinct time periods (45 min, 3 h and 6 h) to permit the recruitment of molecular factors associated with the DNA repair machinery to damaged chromatin sites. Besides analyzing whole cell lysates to determine LAP1 and TRF2 protein levels, nuclear extracts obtained through subcellular fractionation were also included in the analysis, since the enrichment of nuclear factors in these samples would facilitate the detection of alterations in this subset of proteins. According to our results, LAP1 (Figure 4A) and TRF2 (Figure 5A) levels showed a tendency to increase in total protein extracts collected from cells exposed to 200 µg/mL of bleomycin and whose recovery lasted 3 h or 6 h, when compared to the control group. Notably, in these particular conditions, the differences between bleomycin-treated cells and untreated ones were clearly visible in samples of the nuclear protein fraction. Regarding total LAP1 levels, statistically significant increases of approximately 24.4% and 42.4%, respectively, were observed (Figure 4B). Likewise, TRF2 levels augmented by about 18.1% and 46.3%, respectively, showing statistical significance in the latter condition (Figure 5B). Hence, these data suggest that LAP1 and TRF2 protein levels increase in the nucleus within a few hours following DNA damage. Together with the fact that their binding affinity also appears to be superior in this biological context (Figure 3B), one may deduce that these proteins are functionally related during the DDR.

### 3.4. LAP1 and TRF2 Co-Localize at the Nuclear Membrane and Intranuclearly in Response to DNA Damage In Vivo

**Figure 6:**
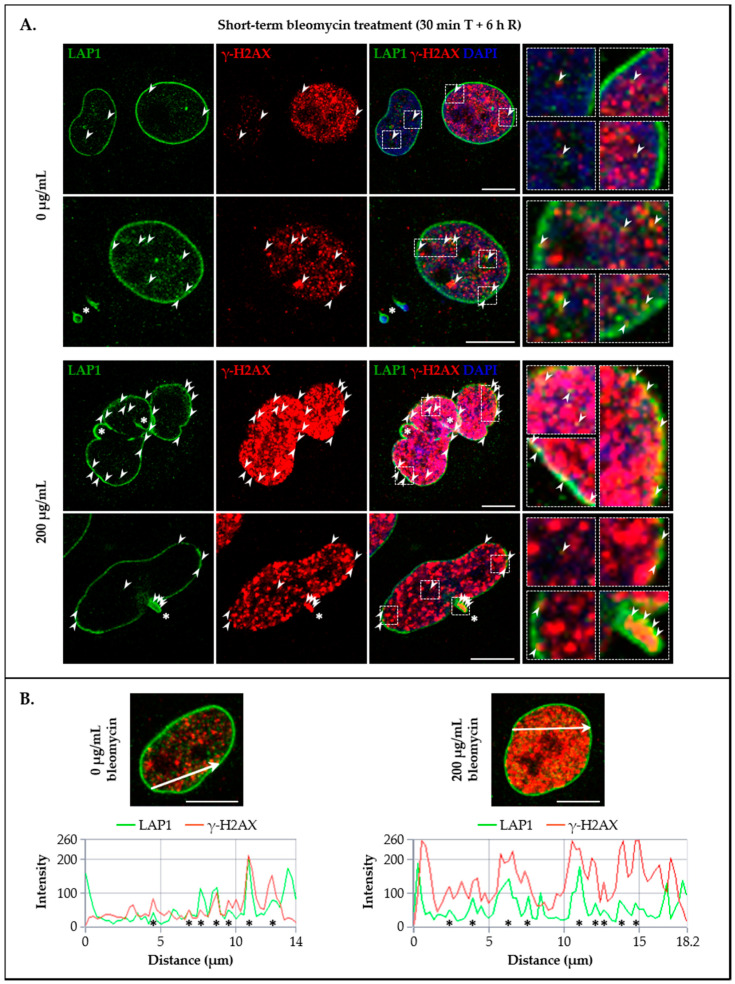
Analysis of the subcellular co-distribution of LAP1 and γ-H2AX in the human HeLa cell line. (**A**) Immunolocalization of LAP1 (green) and γ-H2AX (red) in control conditions and after DNA damage induction through cell exposure to 200 µg/mL of bleomycin for 30 min (T), followed by 6 h of recovery (R). Upon fixation, HeLa cells were immunostained with LAP1- and γ-H2AX-specific primary antibodies linked to AF 488- and AF 594-conjugated secondary antibodies, respectively, and incubated with mounting medium containing DAPI (blue). Image acquisition was performed using an LSM 880 confocal laser scanning microscope with Airyscan. Representative microphotographs (one section in the Z-axis) are shown for each experimental condition. The presence of nuclear LAP1/γ-H2AX co-localization sites are identified by arrowheads and highlighted in the ROIs of merged images (yellow/orange foci). Asterisks denote the presence of LAP1 in micronuclei (second panel), in regions of increased fragility of the NE (third panel) and in a nuclear bleb together with γ-H2AX (fourth panel). Scale bars, 10 µm. (**B**) Confocal microscopic profiles (one section in the Z-axis) representing LAP1 (green) and γ-H2AX (red) fluorescence intensities at a specific nuclear distance (arrow). Asterisks indicate co-localization sites where signal intensity increases for both proteins. Scale bars, 10 µm. γ-H2AX, histone variant H2AX phosphorylated at Ser139; AF, Alexa Fluor; DAPI, 4′,6-diamidino-2-phenylindole; LAP1, lamina-associated polypeptide 1; NE, nuclear envelope; ROI, region of interest.

**Figure 7:**
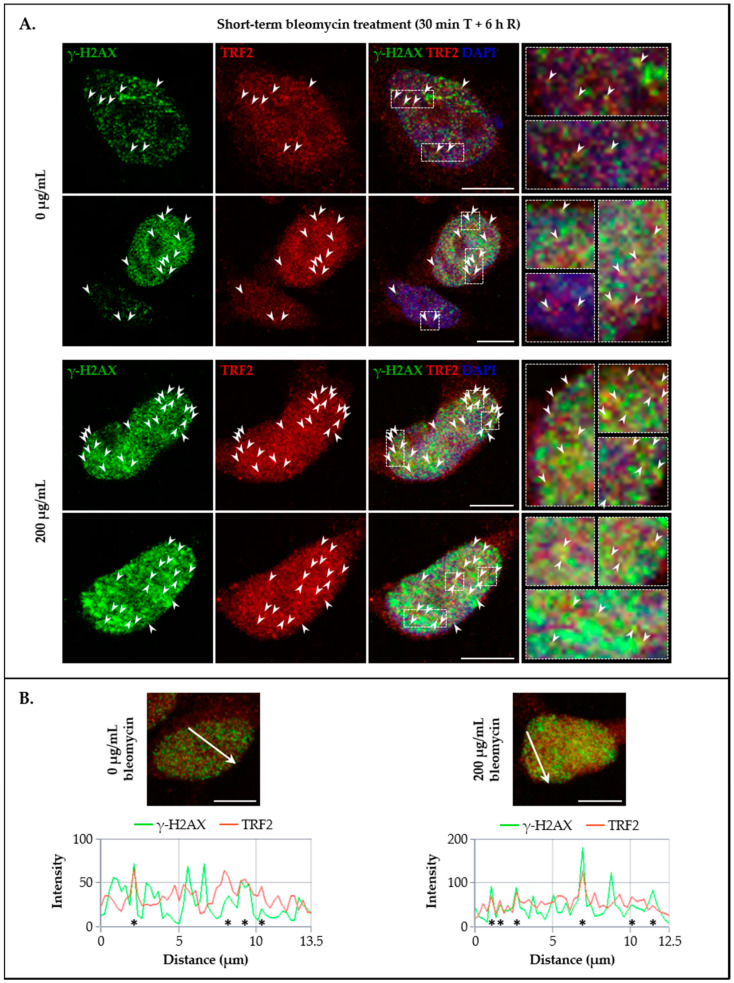
Analysis of the subcellular co-distribution of TRF2 and γ-H2AX in the human HeLa cell line. (**A**) Immunolocalization of γ-H2AX (green) and TRF2 (red) in control conditions and after DNA damage induction through cell exposure to 200 µg/mL of bleomycin for 30 min (T), followed by 6 h of recovery (R). Upon fixation, HeLa cells were immunostained with γ-H2AX- and TRF2-specific primary antibodies linked to AF 488- and AF 594-conjugated secondary antibodies, respectively, and incubated with mounting medium containing DAPI (blue). Image acquisition was performed using an LSM 880 confocal laser scanning microscope with Airyscan. Representative microphotographs (one section in the Z-axis) are shown for each experimental condition. The presence of nuclear TRF2/γ-H2AX co-localization sites are identified by arrowheads and highlighted in the ROIs of merged images (yellow/orange foci). Scale bars, 10 µm. (**B**) Confocal microscopic profiles (one section in the Z-axis) representing γ-H2AX (green) and TRF2 (red) fluorescence intensities at a specific nuclear distance (arrow). Asterisks indicate co-localization sites where signal intensity increases for both proteins. Scale bars, 10 µm. γ-H2AX, histone variant H2AX phosphorylated at Ser139; AF, Alexa Fluor; DAPI, 4′,6-diamidino-2-phenylindole; ROI, region of interest; TRF2, telomeric repeat-binding factor 2.

**Figure 8:**
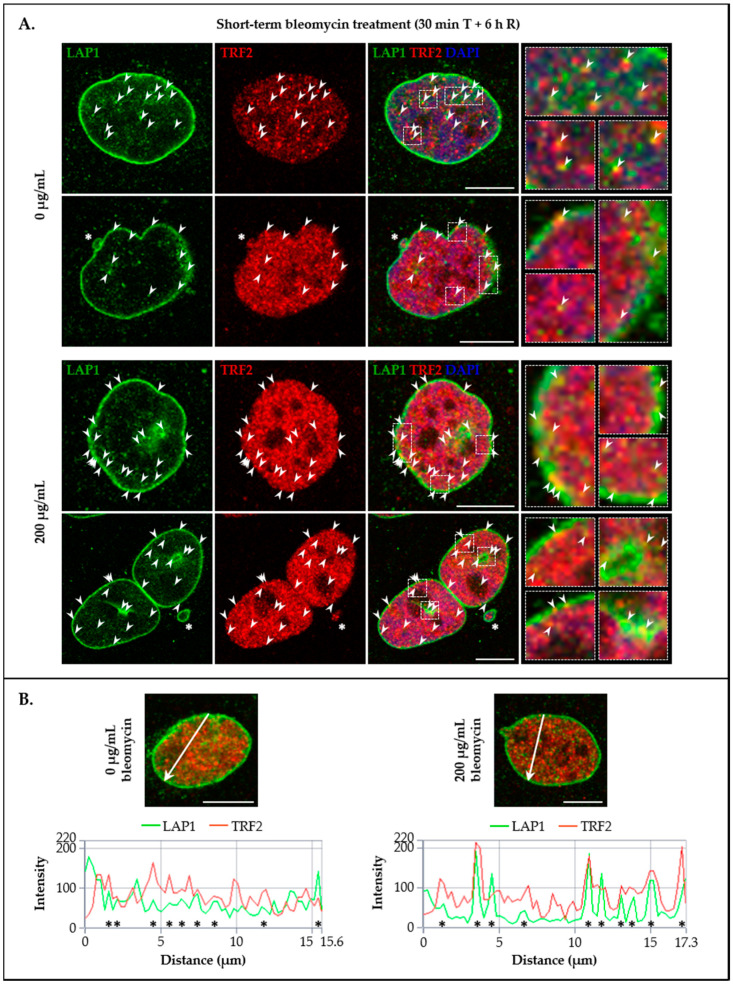
Analysis of the subcellular distribution of LAP1:TRF2 complex in the human HeLa cell line. (**A**) Immunolocalization of LAP1 (green) and TRF2 (red) in control conditions and after DNA damage induction through cell exposure to 200 µg/mL of bleomycin for 30 min (T), followed by 6 h of recovery (R). Upon fixation, HeLa cells were immunostained with LAP1- and TRF2-specific primary antibodies linked to AF 488- and AF 594-conjugated secondary antibodies, respectively, and incubated with mounting medium containing DAPI (blue). Image acquisition was performed using an LSM 880 confocal laser scanning microscope with Airyscan. Representative microphotographs (one section in the Z-axis) are shown for each experimental condition. The presence of nuclear LAP1/TRF2 co-localization sites are identified by arrowheads and highlighted in the ROIs of merged images (yellow/orange foci). Asterisks denote the co-presence of LAP1 and TRF2 in a nuclear bleb (second panel) and in a micronucleus (fourth panel). Scale bars, 10 µm. (**B**) Confocal microscopic profiles (one section in the Z-axis) representing LAP1 (green) and TRF2 (red) fluorescence intensities at a specific nuclear distance (arrow). Asterisks indicate co-localization sites where signal intensity increases for both proteins. Scale bars, 10 µm. AF, Alexa Fluor; DAPI, 4′,6-diamidino-2-phenylindole; LAP1, lamina-associated polypeptide 1; ROI, region of interest; TRF2, telomeric repeat-binding factor 2.

Having verified that LAP1:TRF2 complex formation after DNA damage induction in human cells is accompanied by an increase in the respective protein levels, the next strategy was to characterize their subcellular distribution in vivo. For this purpose, immunocytochemistry assays were performed to investigate the co-localization of endogenous LAP1, TRF2 and γ-H2AX in HeLa cells’ nuclei in a baseline state and upon a short-term bleomycin exposure (200 µg/mL for 30 min, followed by 6 h of recovery). A general analysis by confocal microscopy revealed that all proteins are located at the expected nuclear compartments irrespective of the experimental condition, wherein γ-H2AX (Figure 6A and Figure 7A) and TRF2 (Figure 7A and Figure 8A) are typically dispersed across the nucleoplasm whereas LAP1 is mostly distributed throughout the NE (Figure 6A and Figure 8A), being consistent with previous descriptions for each protein [18,22,44,59,60]. Regarding their fluorescence intensity in the nucleus, the results indicated that, overall, the abundance of the three proteins augments after cell exposure to the DNA-damaging treatment comparatively to the control group (Supplementary Figure S4A,C,E), thus confirming the prior findings (Figure 2C, Figure 4B and Figure 5B). Focusing on the LAP1/γ-H2AX and TRF2/γ-H2AX immunocytochemistry experiments, two major γ-H2AX staining patterns were perceptible, comprising nuclei with discrete, countable foci and nuclei with pan-nuclear staining, which were more frequently encountered in cells cultured under normal conditions or in those incubated with bleomycin, respectively (Figure 6A and Figure 7A), as formerly reported by others [59]. Interestingly, a careful visual examination of the microphotographs permitted the identification of multiple sites juxtaposed to the NE and in the nuclear interior where LAP1 staining overlaps with that of γ-H2AX, not only in bleomycin-treated cells but also in untreated ones that exhibited some degree of DNA damage (Figure 6A (arrowheads)). In fact, this INM protein was detected in the periphery of micronuclei (Figure 6A (asterisk in the second panel)) as well as nuclear blebs (Figure 6A (asterisk in the fourth panel)), in some cases co-existing with γ-H2AX in such structures. Furthermore, we observed that LAP1 accumulates at regions of increased fragility of the nuclear membrane in cells carrying extensive DNA lesions and potentially undergoing programmed cell death (Figure 6A (asterisks in the third panel)). To quantify the co-localization between LAP1 and γ-H2AX, a pixel intensity spatial correlation analysis based on Manders’ (spatial co-occurrence) and Pearson’s (intensity correlation) coefficients was carried out. Essentially, it was found that the fraction of LAP1 that is localized to the same nuclear areas where γ-H2AX is present (40% and 46% in control and bleomycin-treated cells, respectively) is quite similar to the fraction of γ-H2AX that is co-distributed with LAP1 (41% and 44% in control and bleomycin-treated cells, respectively) (Supplementary Figure S4B). It was also evident that the fluorescence intensities of these two overlapping proteins are positively correlated, although there is only a moderate association (0.49 and 0.52 in control and bleomycin-treated cells, respectively) (Supplementary Figure S4B). Moreover, we obtained confocal microscopic profiles at a defined nuclear distance to validate the presence of LAP1/γ-H2AX co-localization points. With this approach, it was possible to confirm that, although considerable proportions of LAP1 and γ-H2AX co-occur over space, the intensity of their corresponding green and red fluorescent signals increases simultaneously in not all but at specific sites (Figure 6B (asterisks)), representing a subset of spots where a yellow color is visible in the merged images (Figure 6A (arrowheads in ROIs)). In light of these data, it is conceivable that LAP1 has a functional role in the DDR process, which is most likely accomplished at such nuclear regions where, besides existing spatial coincidence with γ-H2AX, there is additionally an accumulation of both proteins. In the case of TRF2 and as expected, we visualized that its staining overlaps with the one of γ-H2AX in numerous points throughout the nucleus, including very near to the nuclear boundary, in the two experimental conditions tested (Figure 7A (arrowheads)). The quantitative co-localization analysis showed that the amount of γ-H2AX co-occurring with TRF2 (75% and 83% in control and bleomycin-treated cells, respectively) is higher than the amount of TRF2 located at γ-H2AX-positive nuclear areas (55% and 66% in control and bleomycin-treated cells, respectively) (Supplementary Figure S4D). In addition, we discovered a relatively strong positive correlation between the staining intensities of TRF2 and γ-H2AX (0.74 and 0.78 in control and bleomycin-treated cells, respectively) (Supplementary Figure S4D). The confocal microscopic profiles of TRF2/γ-H2AX fluorescent signals further supported the existence of several co-localization points where both proteins evidence an association in terms of spatial overlap and increased abundance (Figure 7B (asterisks)), which likely correspond to DNA damage sites located outside of telomeres where TRF2 has been shown to concentrate transiently during the DDR [40,41,42,43,44].

With regard to LAP1/TRF2 immunocytochemistry preparations, we observed many microscopically detectable foci where these proteins are co-distributed in the nucleus, occurring in close proximity to the NE and within the nucleoplasm in bleomycin-treated cells and those of the control group (Figure 8A (arrowheads)). Another noteworthy finding is the simultaneous presence of LAP1 and TRF2 in nuclear blebs (Figure 8A (asterisk in the second panel)) and micronuclei (Figure 8A (asterisk in the fourth panel)). It appears that the co-localization between the two proteins takes place at damaged chromatin sites, which are particularly abundant after cell exposure to bleomycin, but can be found in a baseline state as well (Figure 6A and Figure 7A). This assumption is supported by the fact that, similarly to TRF2 (Figure 7A (arrowheads)), LAP1 co-localizes with γ-H2AX, namely in the abovementioned abnormal nuclear structures associated with genomic damage (Figure 6A (arrowheads, asterisks)). Therefore, the presented results point to the in vivo LAP1:TRF2 complex formation as a cellular response to DNA breaks, as already validated by co-immunoprecipitation (Figure 3B), and additionally show its subcellular distribution. Moreover, based on a fluorescence co-occurrence analysis, we determined that the fraction of LAP1 existing in nuclear areas containing TRF2 (66% and 67% in control and bleomycin-treated cells, respectively) is slightly superior to the fraction of TRF2 that spatially overlaps with LAP1 (57% and 59% in control and bleomycin-treated cells, respectively) (Supplementary Figure S4F). In terms of signal intensity correlation, a relatively strong positive relationship between these proteins was found (0.7 in control and bleomycin-treated cells) (Supplementary Figure S4F), suggestive of protein attraction. In line with this, the confocal microscopic profiles confirmed that, in specific nuclear regions, LAP1 and TRF2 not only co-distribute over space but also associate in terms of fluorescence intensity with each other, occasionally showing a proportional staining pattern (Figure 8B (asterisks)). This is clearly perceptible by the presence of bright yellow foci resulting from the combined contribution of LAP1′s green and TRF2′s red signals in the superimposed images (Figure 8A (arrowheads in ROIs)).

**Figure 9:**
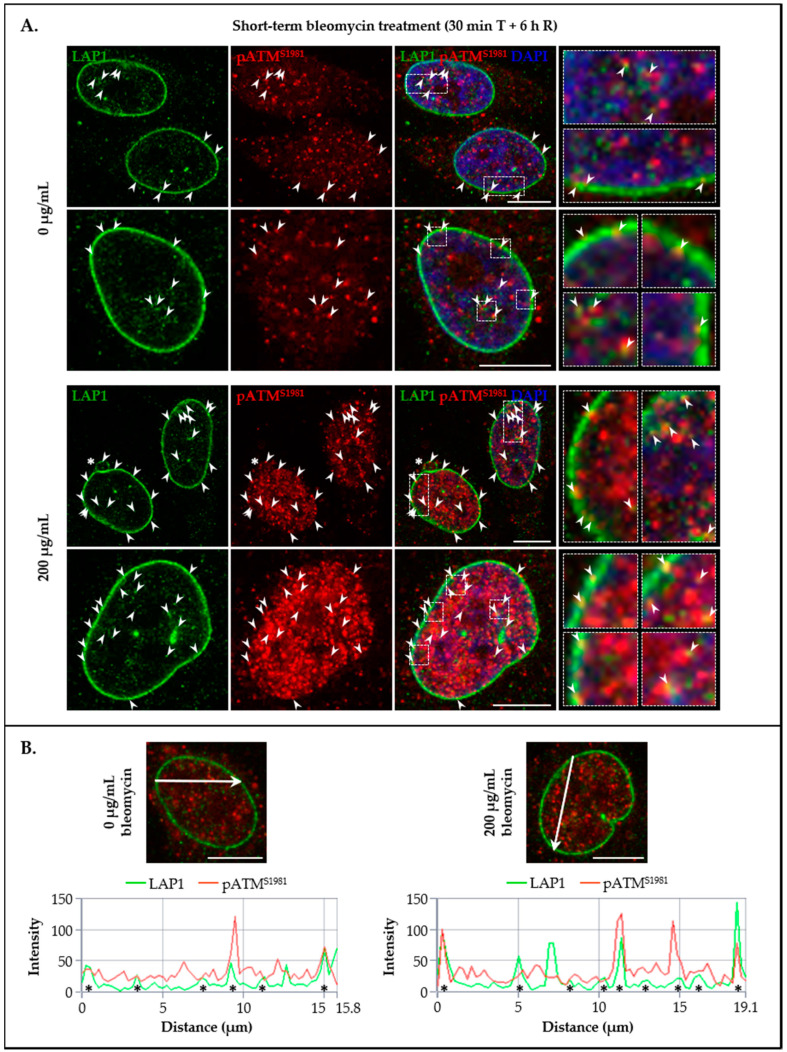
Analysis of the subcellular co-distribution of LAP1 and pATM^S1981^ in the human HeLa cell line. (**A**) Immunolocalization of LAP1 (green) and pATM^S1981^ (red) in control conditions and after DNA damage induction through cell exposure to 200 µg/mL of bleomycin for 30 min (T), followed by 6 h of recovery (R). Upon fixation, HeLa cells were immunostained with LAP1- and pATM^S1981^-specific primary antibodies linked to AF 488- and AF 594-conjugated secondary antibodies, respectively, and incubated with mounting medium containing DAPI (blue). Image acquisition was performed using an LSM 880 confocal laser scanning microscope with Airyscan. Representative microphotographs (one section in the Z-axis) are shown for each experimental condition. The presence of nuclear LAP1/pATM^S1981^ co-localization sites are identified by arrowheads and highlighted in the ROIs of merged images (yellow/orange foci). The asterisk denotes the presence of LAP1 in a nuclear bleb together with pATM^S1981^ (third panel). Scale bars, 10 µm. (**B**) Confocal microscopic profiles (one section in the Z-axis) representing LAP1 (green) and pATM^S1981^ (red) fluorescence intensities at a specific nuclear distance (arrow). Asterisks indicate co-localization sites where signal intensity increases for both proteins. Scale bars, 10 µm. AF, Alexa Fluor; ATM, ataxia–telangiectasia mutated protein; DAPI, 4′,6-diamidino-2-phenylindole; LAP1, lamina-associated polypeptide 1; pATM^S1981^, ATM phosphorylated at Ser1981; ROI, region of interest.

**Figure 10:**
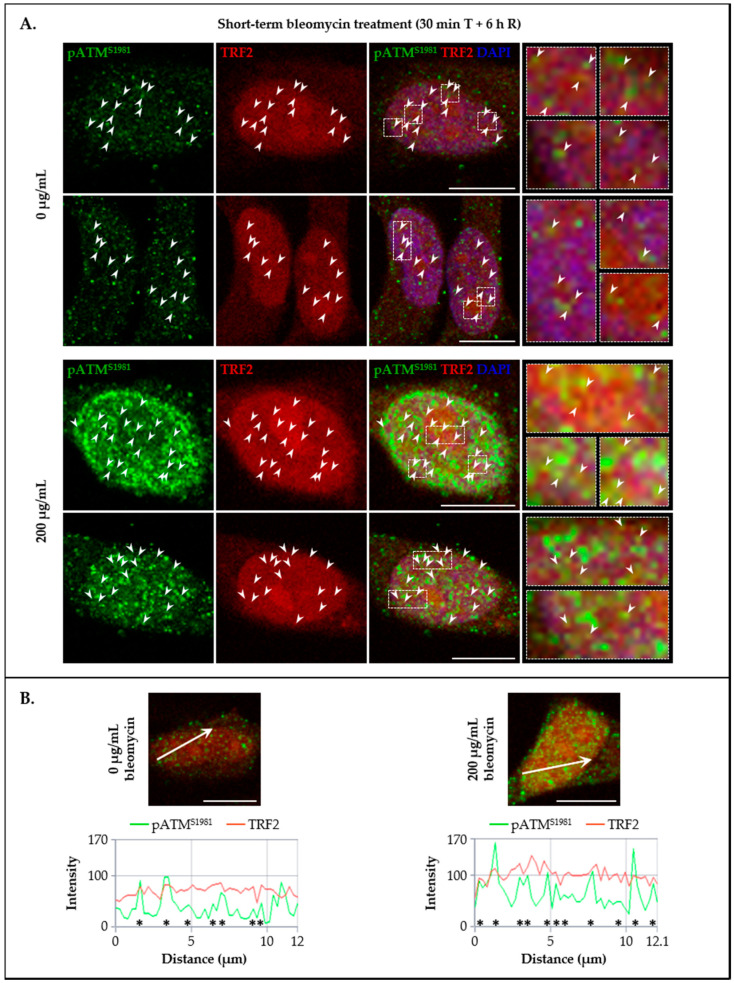
Analysis of the subcellular co-distribution of TRF2 and pATM^S1981^ in the human HeLa cell line. (**A**) Immunolocalization of pATM^S1981^ (green) and TRF2 (red) in control conditions and after DNA damage induction through cell exposure to 200 µg/mL of bleomycin for 30 min (T), followed by 6 h of recovery (R). Upon fixation, HeLa cells were immunostained with pATM^S1981^- and TRF2-specific primary antibodies linked to AF 488- and AF 594-conjugated secondary antibodies, respectively, and incubated with mounting medium containing DAPI (blue). Image acquisition was performed using an LSM 880 confocal laser scanning microscope with Airyscan. Representative microphotographs (one section in the Z-axis) are shown for each experimental condition. The presence of nuclear TRF2/pATM^S1981^ co-localization sites are identified by arrowheads and highlighted in the ROIs of merged images (yellow/orange foci). Scale bars, 10 µm. (**B**) Confocal microscopic profiles (one section in the Z-axis) representing pATM^S1981^ (green) and TRF2 (red) fluorescence intensities at a specific nuclear distance (arrow). Asterisks indicate co-localization sites where signal intensity increases for both proteins. Scale bars, 10 µm. AF, Alexa Fluor; ATM, ataxia–telangiectasia mutated protein; DAPI, 4′,6-diamidino-2-phenylindole; pATM^S1981^, ATM phosphorylated at Ser1981; ROI, region of interest; TRF2, telomeric repeat-binding factor 2.

To further explore the potential in vivo co-localization of LAP1 and TRF2 with other DDR factors in addition to γ-H2AX, their subcellular distribution together with pATM^S1981^ in HeLa cells’ nuclei was investigated by immunocytochemistry in the same experimental conditions mentioned above. Of note, this ATM’s serine residue is rapidly subjected to autophosphorylation in response to DNA damage and is essential for the stable association of this kinase with DSBs, as well as for its activation and subsequent ATM-mediated phosphorylation events in an early stage of the DDR process [31,61]. Resembling LAP1/γ-H2AX (Figure 6A), TRF2/γ-H2AX (Figure 7A) and LAP1/TRF2 (Figure 8A) immunostainings, we detected the simultaneous presence of pATM^S1981^ and either LAP1 (Figure 9A (arrowheads)) or TRF2 (Figure 10A (arrowheads)) in discrete nuclear sites located both peripherally and more internally, in particular in bleomycin-treated cells and, to a lesser extent, in untreated ones as well. Interestingly, LAP1 was also found to overlap with pATM^S1981^ in nuclear blebs’ margin (Figure 9A (asterisk in the third panel)), as already observed when studying its co-occurrence with the other two proteins (Figure 6A and Figure 8A (asterisks)). Furthermore, as evidenced by the confocal microscopic profiles, several points where an increase in LAP1′s (Figure 9B (asterisks)) or TRF2′s (Figure 10B (asterisks)) fluorescent signals was accompanied by an augmented fluorescence intensity of pATM^S1981^ could be identified in the nucleus.

In essence, the main conclusions taken from this set of experiments are that: (i) LAP1 and TRF2 partially co-localize with damaged chromatin-associated factors γ-H2AX and pATM^S1981^ in intranuclear regions as well as in close proximity to the inner surface of the NE in human cells; (ii) LAP1 occasionally co-occurs with TRF2, γ-H2AX and pATM^S1981^ in nuclear blebs and micronuclei; and (iii) LAP1 and TRF2 concentrations increase simultaneously at discrete focal points throughout the nucleus, where they most likely are bound together at DNA damage sites, which anticipates a cooperative role for these proteins in the DDR.

## 4. Discussion

In the last decade, a phenotypic association of LAP1 deficiency with severe cardiac, muscular and neurological pathologies was uncovered [9,10,11,12,13,14,15]. To date, however, the physiological properties of human LAP1 are not well understood, prompting us to investigate novel functions for this protein. Based on the suggestion of a new regulatory role in the DDR process that emerged from a recent LAP1 interactome study [29], along with the previous description of an interaction between LAP1 and TRF2 [47]—a protein with documented involvement in DNA damage repair [40,41,42,43,44,45,46], we hypothesized that LAP1 might be functionally associated with TRF2 in a biological context of DDR signaling activation.

In this report, human LAP1:TRF2 complex formation was first validated using in vitro and in vivo techniques. Through a blot overlay assay, it was demonstrated that both known LAP1B variants (i.e., full-length LAP1B and the shorter variant LAP1B ΔA185) interact directly with TRF2 in vitro (Figure 1B). Notwithstanding that additional work is required to define the exact TRF2-binding motif in LAP1, one can presume, considering the predicted membrane topology of this INM protein [16,17,18], that it is included in the N-terminal nucleoplasmic domain, which has already been shown to communicate with other intranuclear proteins (e.g., PP1) [22]. To gain insights into the physiological cellular processes in which the LAP1:TRF2 complex could be biologically relevant in vivo, LAP1 co-immunoprecipitation assays were carried out following DNA-damaging H_2_O_2_ and bleomycin treatments. Using HeLa cells as a human model, we found that LAP1 interaction with TRF2 is established in response to the induction of DNA breaks (Figure 3B). We also tested the association of these proteins in HeLa and SH-SY5Y cells in control conditions, but, in this case, the co-immunoprecipitation experiments did not reveal the presence of the LAP1:TRF2 complex (Figure 3A and Supplementary Figure S1A). These results contrast with those from the study that originally proposed a link between both proteins in a physiological context. In that work, a protein–protein interaction screening strategy based on yellow fluorescent protein (YFP) complementation was employed to identify novel regulators of shelterin complex proteins. Given the increased ability of this technique to detect transient protein interactions as compared to the co-immunoprecipitation procedure [47], we suspect that our approach may have not captured the LAP1:TRF2 association in untreated cells. The most probable scenario is, in fact, that this interaction also occurs under normal conditions, considering that, even in a baseline state, cells are continuously exposed to endogenous sources of DNA insults (e.g., metabolic products, namely ROS) [62]. As such, the negative outcome of these co-immunoprecipitation assays is probably explained by the relatively low levels of DNA damage in control cells, which may be insufficient to amplify the interaction of LAP1 with TRF2 to a level that is beyond the detection limit of our technique. Overall, the data presented here suggest that the physical association between the two proteins may not have a constitutive nature; instead, it most likely requires specific stimulation by the existence of DNA lesions, so that its transitory occurrence is triggered within the cell, thus explaining the success of the co-immunoprecipitation experiments conducted in a context of exposure to genotoxic agents. It should be mentioned that, building on the prior observation that LAP1 also binds to RAP1 [47], we searched for this protein interaction under conditions of DNA damage induction in HeLa cells. Nevertheless, the LAP1 co-immunoprecipitation results did not validate such an association (Supplementary Figure S2B), indicating that RAP1 may not be part of a putative tricomplex with LAP1 and TRF2 in this particular context. As described before, TRF2 plays a dual role in the DDR. On the one hand, it inhibits this signaling cascade at chromosome ends to maintain their structure, which is accomplished, in part, by working together with the binding partner RAP1 [38,39]. On the other hand, TRF2 migrates transiently to extra-telomeric regions harboring damaged chromatin, where it activates DNA repair pathways [40,41,42,43,44], but RAP1 does not appear to follow the same fate [42]. Another aspect worth referencing is that RAP1 has been shown to increase the binding affinity of TRF2 for telomeric DNA while reducing its attraction to double-stranded DNA [63,64]. Hence, one can deduce, from the absence of RAP1 in the co-immunoprecipitates obtained after cell exposure to H_2_O_2_, that the protein complex comprising LAP1 and TRF2 is functionally active at chromatin sites located outside of telomeres, being potentially involved in the repair of broken DNA ends.

Having in mind that many reversible post-translational modifications (e.g., phosphorylation, ubiquitination, SUMOylation, PARylation, methylation and acetylation) allow for the rapid, temporary regulation of the function, structure and/or localization of DDR factors [30,62], the next objective was to characterize the molecular mechanisms controlling the LAP1:TRF2 interaction in vivo. As previously explained, we decided to focus on the process of phosphorylation because both proteins possess several serine and/or threonine residues in their sequences that are phosphorylated by ATM/ATR in response to DNA damage [41,48]. It is also important to note that LAP1 [65,66] and TRF2 [67] have been identified in phosphatase interactome studies as putative PP1-binding proteins and, in the case of LAP1, PP1 has already been functionally implicated in its dephosphorylation on two serine residues [19]. To test the impact of modulating phosphorylation on the direct binding of LAP1 to TRF2, a LAP1 co-immunoprecipitation assay was performed in HeLa cells subjected to an okadaic acid treatment that inhibits the activity of PP1 and PP2A, leading to increased phosphorylation levels of both LAP1B and LAP1C according to prior studies [19,22]. Essentially, we uncovered that the physical association between LAP1 and TRF2 occurs preferentially when LAP1 is phosphorylated (Figure 3C)—and perhaps TRF2 as well if it proves to be a PP1 substrate—, herein mimicked experimentally through phosphatase activity inhibition. These findings can be translated to a natural environment of DDR signaling activation, wherein this increase in LAP1 phosphorylation state is likely dependent on ATM/ATR kinase activity. Moreover, our data raise the possibility that PP1-mediated LAP1 dephosphorylation may control the disassembly of this transient protein complex, presumably upon the resolution of genomic DNA breaks. Of interest, besides providing a tool for studying (de)phosphorylation-regulated events, okadaic acid has been found to induce oxidative DNA damage in human cells [68,69], which gives additional support to our earlier conclusion that the in vivo occurrence of the LAP1:TRF2 complex is elicited intracellularly by the presence of DNA lesions and, more importantly, allows for the establishment of a link between this protein interaction, the DDR process and the regulatory mechanism of reversible phosphorylation. Further validation for the above results was obtained by HPLC–MS analysis of the two protein bands containing immunoprecipitated LAP1 from okadaic acid-treated SH-SY5Y cells, which permitted the identification of two peptides that align with the known sequence of human TRF2 (Table 1 and Supplementary Figure S3B), thus confirming that LAP1 binding to TRF2 is regulated by phosphorylation. This post-translational modification may provide a trigger to the interaction of LAP1 with TRF2 in response to genotoxic insults, either by exerting a positive modulatory effect on its binding affinity for TRF2 and/or by stimulating its recruitment to DNA breaks. In accordance with this hypothesis, it has been demonstrated that the TRF2 protein phosphorylated at Thr230 (numbering relative to TRF2′s NCBI reference sequence: NP_005643.2 [50]) is dissociated from telomeric DNA and concentrates at damaged chromatin sites, which may favor its association with other DNA repair mediators [41], among which LAP1 is putatively included. Although the functional relevance of ATM/ATR-mediated LAP1 phosphorylation is currently unclear, it is noteworthy that all of the identified DNA damage-responsive phosphorylation sites are located in a region of the N-terminal nucleoplasmic domain common to both LAP1B and LAP1C (Supplementary Figure S3A), suggesting that the two human LAP1 isoforms may participate in the DDR.

Following the in vivo validation of the LAP1:TRF2 complex and its regulation by phosphorylation, the endogenous levels of these proteins were quantified by immunoblotting in HeLa cells exposed to DNA-damaging concentrations of bleomycin. We noticed that, after a short-term treatment and subsequent recovery phase, total LAP1 and TRF2 levels are significantly augmented in nuclear lysates relative to the control group (Figure 4B and Figure 5B, respectively), an alteration that accompanies the generation of DNA lesions, as assessed by the concomitant increase in γ-H2AX protein levels (Figure 2C). In line with this, a quantitative fluorescence analysis of immunostained LAP1, TRF2 and γ-H2AX revealed an overall accumulation of these proteins in the nucleus of bleomycin-treated cells in comparison with control ones (Supplementary Figure S4A,C,E). The visual examination of confocal microphotographs provided additional information regarding the subcellular distribution of the LAP1:TRF2 interaction in vivo. Through the immunolocalization of both proteins, we showed that they co-localize in focal points near the INM and in the nuclear interior (Figure 8A (arrowheads)), which represent the natural environments where LAP1 and TRF2, respectively, are primarily expressed. The fact that LAP1 forms a complex with TRF2 juxtaposed to the inner side of the NE is consistent with our assumption that the direct interaction of LAP1 with TRF2, validated by the in vitro blot overlay assay (Figure 1B), is mediated by its nucleoplasmic domain. Furthermore, the nuclear co-distribution of these proteins with the DDR factors γ-H2AX and pATM^S1981^ was investigated to confirm their spatial concentration at DNA damage sites. Besides the expected co-occurrence of TRF2 with γ-H2AX (Figure 7A (arrowheads)) and pATM^S1981^ (Figure 10A (arrowheads)), a result that is in agreement with the previously described transitory migration of TRF2 to non-telomeric DNA breaks [40,41,42,43,44], it was found that LAP1 is partially localized to γ-H2AX-positive (Figure 6A (arrowheads)) and pATM^S1981^-positive (Figure 9A (arrowheads)) sites as well. In fact, a very similar staining pattern is observed when comparing LAP1/γ-H2AX, TRF2/γ-H2AX, LAP1/TRF2, LAP1/pATM^S1981^ and TRF2/pATM^S1981^ immunocytochemistry assays, with numerous co-localization points being detected at the nuclear periphery and intranuclearly in cells incubated with bleomycin and in untreated ones carrying basal levels of DNA lesions (Figure 6A, Figure 7A, Figure 8A, Figure 9A and Figure 10A (arrowheads), respectively). Remarkably, LAP1 is additionally present in nuclear blebs and micronuclei, usually in co-existence with γ-H2AX (Figure 6A (asterisk in the fourth and second panels, respectively)), TRF2 (Figure 8A (asterisk in the second and fourth panels, respectively)) and pATM^S1981^ (Figure 9A (asterisk in the third panel)). Taken together, our data strongly support the occurrence of the LAP1:TRF2 complex at DNA damage-associated γ-H2AX foci dispersed throughout the nuclear compartments in human cells. It is widely acknowledged that the local production of γ-H2AX at broken DNA ends and its subsequent spreading into large contiguous chromatin regions allows for the early amplification of DDR signals, maximizing the mobilization of multiple repair factors to DNA lesions, where a network of protein–protein interactions is established [58,62]. Accordingly, one can envisage that LAP1 and TRF2 may be individually attracted to such γ-H2AX-enriched areas, which, in turn, brings the two proteins into close proximity, thus potentiating their direct association to assemble the LAP1:TRF2 complex and its binding to damaged chromatin. Regarding TRF2, a recent report proposed a two-step recruitment mechanism, wherein, in a first stage, this protein rapidly and temporarily migrates to DNA damage sites located outside of telomeres in a poly(ADP-ribose) polymerase (PARP)-dependent manner, followed by its stable and more prolonged accumulation at those regions in a second phase mediated by the MRE11–RAD50–NBS1 (MRN) complex [44]. In the case of LAP1, we speculate that ATM/ATR-dependent reversible phosphorylation may be responsible for its retention at γ-H2AX-associated DSBs, for instance by inducing conformational changes in LAP1 and/or by reducing its interaction with the nuclear lamina that normally contributes to its positioning at the INM, which would make possible for this protein to shift spatially from its routine NE location to the nucleoplasm. Our observation that LAP1 co-localizes with pATM^S1981^ appears to give some validation to this hypothesis, having in mind that autophosphorylation of ATM at Ser1981 is not only required for its localization to DNA damage sites but also for its ability to phosphorylate downstream DDR substrates [61]. Of note, the observed increase in the nuclear abundance of LAP1 within a few hours upon the infliction of DNA lesions (Figure 4B and Supplementary Figure S4A,E) may be suggestive of de novo protein synthesis and/or decreased protein degradation, possibly related with the necessity to compensate for its recruitment from the INM to sites of DNA breaks in the nuclear interior in order to maintain NE structural integrity. This also holds true for TRF2 (Figure 5B and Supplementary Figure S4C,E) because, while a fraction of this protein is transiently involved in extra-telomeric DNA repair functions, a sufficient amount of TRF2 must remain attached to the chromosome ends to ensure their efficient capping and protection. In accordance with our findings, previous studies showed evidences of an upregulation of the mRNA levels of both LAP1 [70] and TRF2 [71] following DNA damage in human cells. In addition, a quantitative co-localization analysis was conducted to measure the spatial coincidence and intensity correlation of LAP1/γ-H2AX, TRF2/γ-H2AX and LAP1/TRF2 immunostainings. Based on these results, the fraction of γ-H2AX that overlaps with TRF2 is higher than the fraction that co-distributes with LAP1 and the relationship between the fluorescent signals of these proteins is also stronger for the TRF2/γ-H2AX pair than for the LAP1/γ-H2AX pair (Supplementary Figure S4B,D). This may indicate that TRF2 has an increased propensity for accumulating at DNA damage foci in comparison with LAP1, which agrees with the fact that TRF2 shows affinity for binding directly to DNA. Concerning LAP1/TRF2 co-localization, their fluorescence intensities reveal a relatively strong positive correlation (Supplementary Figure S4F), consistent with these proteins being functional partners.

In conclusion, we reported here, for the first time, the involvement of human LAP1 in the DDR and our work suggests that its biological role in this cellular process, although not yet elucidated, may be intimately associated with the establishment of a protein complex with TRF2 at chromatin regions harboring DNA lesions. Even though some questions remain to be answered and should be investigated in the future to fully understand the contribution of LAP1:TRF2 interaction to DNA damage resolution, it is tempting to propose a hypothetical model for the cooperative role of these proteins in this particular physiological context. The fact that the LAP1:TRF2 complex is spatially distributed throughout distinct nuclear compartments (i.e., inner surface of the NE and nuclear interior) may indicate that each location is associated with a specific function in the DDR process. It is recognized that DNA lesions induced in highly compacted heterochromatin, which is typically distributed at the nuclear periphery, in specific chromosomal regions (e.g., telomeres) and around nucleoli, are not as easily accessible by DDR factors as those localized to less condensed, intranuclear euchromatin and, thus, are more difficult to repair [62,72]. In fact, it has been described that, whereas DNA breaks occurring in the nuclear interior or at the NPCs can be repaired by HR or NHEJ, the ones arising within chromatin domains associated with the INM/nuclear lamina are refractory to repair by HR and, instead, NHEJ or alternative end-joining pathways take action [73]. We postulate that the LAP1:TRF2 interaction established at the nucleoplasm is required for TRF2 accomplishing its known HR-related DNA repair functions [44,45], wherein LAP1, through a yet uncharacterized mechanism, may help stabilizing the association of TRF2 with the non-telomeric injured DNA, allowing for an efficient activation of HR signaling events. In turn, the formation of a protein complex between LAP1 and TRF2 at the vicinity of the INM may provide a means of increasing the local concentration of TRF2 at heterochromatin sites near the NE containing persistent DNA lesions, where their repair may result from the stimulation of NHEJ, a process in which TRF2 has also been proposed to play a role [46]. Additionally, considering a scenario wherein inefficiently repaired DNA or excessive amounts of DNA lesions accumulate in the nucleus, we speculate that LAP1 may facilitate their nuclear export and subsequent degradation in an attempt to prevent genomic instability, as suggested by its presence in the periphery of DNA damage-associated nuclear blebs and micronuclei. Consistent with this hypothesis, the NE protein lamin A/C has recently been implicated in the process of nucleophagy in response to the induction of DNA breaks [74]. Overall, as proposed earlier for other LAP1-interacting partners (e.g., emerin and torsinA), the binding of LAP1 to TRF2 may also have the functional purpose of modulating the activity and/or subcellular positioning of the latter, which would make of LAP1 a key regulatory factor in a broad range of nuclear processes.

## 5. Conclusions

The work presented here provides evidence that the INM protein LAP1 interacts directly with the shelterin complex protein TRF2 and that LAP1:TRF2 complex formation at DSBs is integrated in a specific response of cells against DNA damage. Given that the DDR is a biologically important process that ensures the maintenance of cellular homeostasis and prevents human disease, our study sheds light on a potential mechanism that may be deregulated in *TOR1AIP1*-associated disorders, such as cardiomyopathy and muscular dystrophy, and encourages a more detailed investigation of the physiological functions of this essential INM protein, including the effects of its deficiency on DDR dynamics.

## Figures and Tables

**Table 1:** HPLC–MS-based identification of TRF2 as a LAP1-interacting protein in vivo after inhibition of serine/threonine-specific PP1/PP2A phosphatase activity in the human SH-SY5Y cell line. Whole cell lysates obtained in normal conditions or after okadaic acid treatment (500 nM for 3 h) were immunoprecipitated using a LAP1-specific antibody conjugated to Dynabeads Protein G, followed by SDS–PAGE and HPLC–MS analysis of LAP1B and LAP1C protein bands (≈ 68 and 56 kDa, respectively). Unique LAP1 and TRF2 peptides identified in each experimental condition are listed.

	LAP1B + LAP1C bands (68 + 56 kDa)	
	IP LAP1 + 0 nM OA	IP LAP1 + 500 nM OA
**LAP1 Peptides**		
EGWGVYVTPR		X
LAPQNGGSSDAPAYR		X
FSDEPPEVYGDFEPLVAK		X
LQQQHSEQPPLQPSPVMTR		X
DSHSSEEDEASSQTDLSQTISK	X	X
DSHSSEEDEASSQTDLSQTISKK	X	X
SIQEAPVSEDLVIR	X	X
VNFSEEGETEEDDQDSSHSSVTTVK	X	X
SSSQYIESFWQSSQSQNFTAHDK		X
QPSVLSSGYQK		X
TPQEWAPQTAR	X	
MQNDSILKSELGNQSPSTSSR	X	X
QVTGQPQNASFVK	X	X
QVTGQPQNASFVKR	X	X
SQPAILLLTAAR	X	X
IDGTDKATQDSDTVKLEVDQELSNGFK	X	X
ATQDSDTVKLEVDQELSNGFK	X	X
LEVDQELSNGFK	X	X
FESFPAGSTLIFYK	X	X
DVALVLTVLLEEETLGTSLGLK	X	X
ISHLVLPVQPENALK	X	X
ISHLVLPVQPENALKR	X	X
**TRF2 Peptides**		
NDLLNIIR		X
DLVLPTQALPASPALK		X

HPLC–MS, high-performance liquid chromatography–mass spectrometry; IP, immunoprecipitation; LAP1, lamina-associated polypeptide 1; OA, okadaic acid; PP1/2A, protein phosphatase 1/2A; SDS–PAGE, sodium dodecyl sulfate–polyacrylamide gel electrophoresis; TRF2, telomeric repeat-binding factor 2.

## Supplementary Materials

The following are available online at https://www.mdpi.com/2073-4409/9/8/1804/s1: Supplementary Figure S1: Analysis of in vivo protein interactions between LAP1, TRF2 and RAP1 in the human SH-SY5Y cell line; Supplementary Figure S2: Analysis of in vivo protein interactions between LAP1 and RAP1 in the human HeLa cell line; Supplementary Figure S3: Alignment of LAP1 and TRF2 peptides, identified by HPLC–MS after LAP1 co-immunoprecipitation in the human SH-SY5Y cell line, with the corresponding protein sequences; Supplementary Figure S4: Analysis of co-localization between LAP1, TRF2 and γ-H2AX in the human HeLa cell line.
