# Supplementary material for: Nuclear Accumulation of LAP1:TRF2 Complex during DNA Damage Response Uncovers a Novel Role for LAP1
Source: Cells. 2020 Jul 29;9(8):1804. doi: 10.3390/cells9081804 (PMC7465990; doi:10.3390/cells9081804)
Supplement: Supplementary file 1 [file cells-09-01804-s001.pdf]

cells

Article

Nuclear Accumulation of LAP1:TRF2 Complex During DNA Damage Response Uncovers a Novel Role for LAP1

Cátia D. Pereira, Filipa Martins, Mariana Santos, Thorsten Müeller, Odete A. B. da Cruz e Silva and Sandra Rebelo

Supplementary Materials

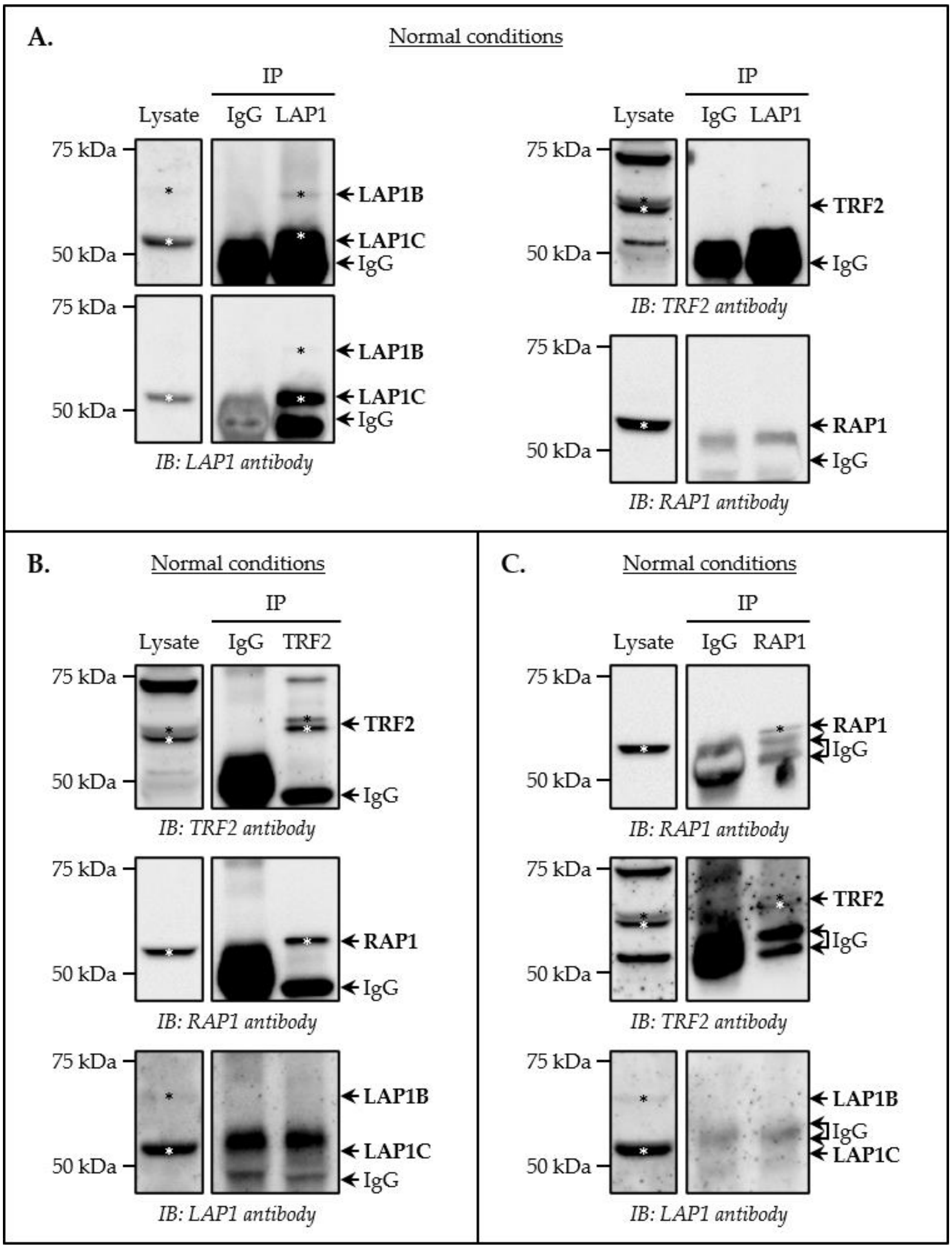

Supplementary Figure 1. Analysis of *in vivo* protein interactions between LAP1, TRF2 and RAP1 in the human SH-SY5Y cell line. (**A**) LAP1 co-immunoprecipitation assay in control conditions. (**B**) TRF2 co-immunoprecipitation assay in control conditions. (**C**) RAP1 co-immunoprecipitation assay in control

conditions. In these experiments, SH-SY5Y cells' whole lysates were immunoprecipitated using specific antibodies against LAP1, TRF2 and RAP1 bound to Dynabeads Protein G. The corresponding negative controls were performed by incubating total protein extracts with Dynabeads Protein G-conjugated rabbit IgG (A) or mouse IgG (B, C). The presence of LAP1, TRF2 and RAP1 in the immunoprecipitates (right), as well as in the initial whole cell lysates (left), was analyzed by immunoblotting using specific antibodies. Asterisks indicate visible LAP1B (≈ 68 kDa), LAP1C (≈ 56 kDa), TRF2 (≈ 69/65 kDa) and RAP1 (≈ 56 kDa) protein bands in each blot. IB, immunoblotting; IgG, immunoglobulin G; IP, immunoprecipitation; LAP1, lamina-associated polypeptide 1; RAP1, repressor/activator protein 1; TRF2, telomeric repeat-binding factor 2.

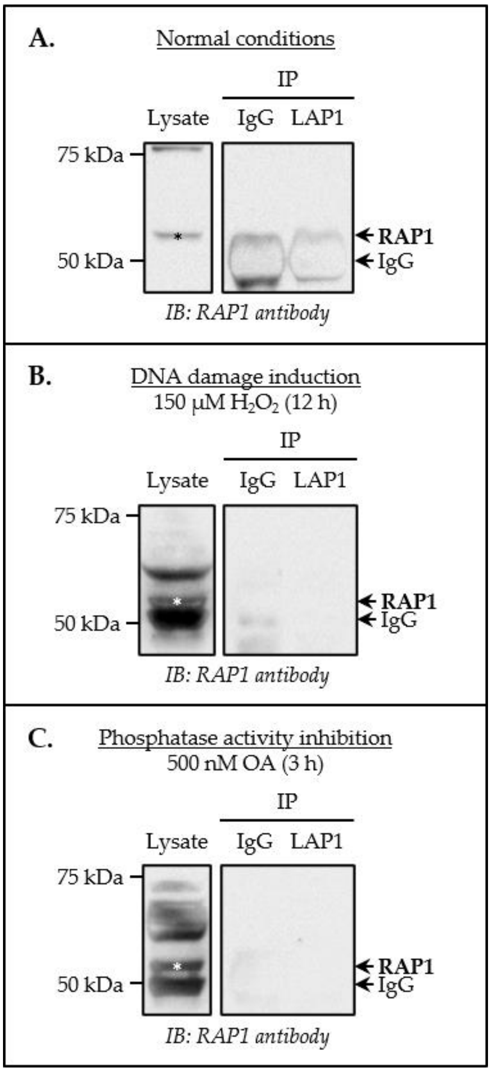

Supplementary Figure 2. Analysis of *in vivo* protein interactions between LAP1 and RAP1 in the human HeLa cell line. (**A**) LAP1 co-immunoprecipitation assay in control conditions. (**B**) LAP1 co-immunoprecipitation assay after cell exposure to H_2O_2 (150 μM for 12 h). (**C**) LAP1 co-immunoprecipitation assay after cell exposure to okadaic acid (500 nM for 3 h). In all assays, HeLa cells' whole lysates were immunoprecipitated using a specific antibody against LAP1 bound to Dynabeads Protein G. The corresponding negative controls were performed by incubating total protein extracts with Dynabeads Protein G-conjugated rabbit IgG. The presence of RAP1 in the immunoprecipitates (right), as well as in the initial whole cell lysates (left), was analyzed by immunoblotting using a specific antibody. Asterisks indicate the visible RAP1 (≈ 56 kDa) protein band in each blot. H_2O_2, hydrogen peroxide; IB, immunoblotting; IgG, immunoglobulin G; IP, immunoprecipitation; LAP1, lamina-associated polypeptide 1; OA, okadaic acid; RAP1, repressor/activator protein 1.

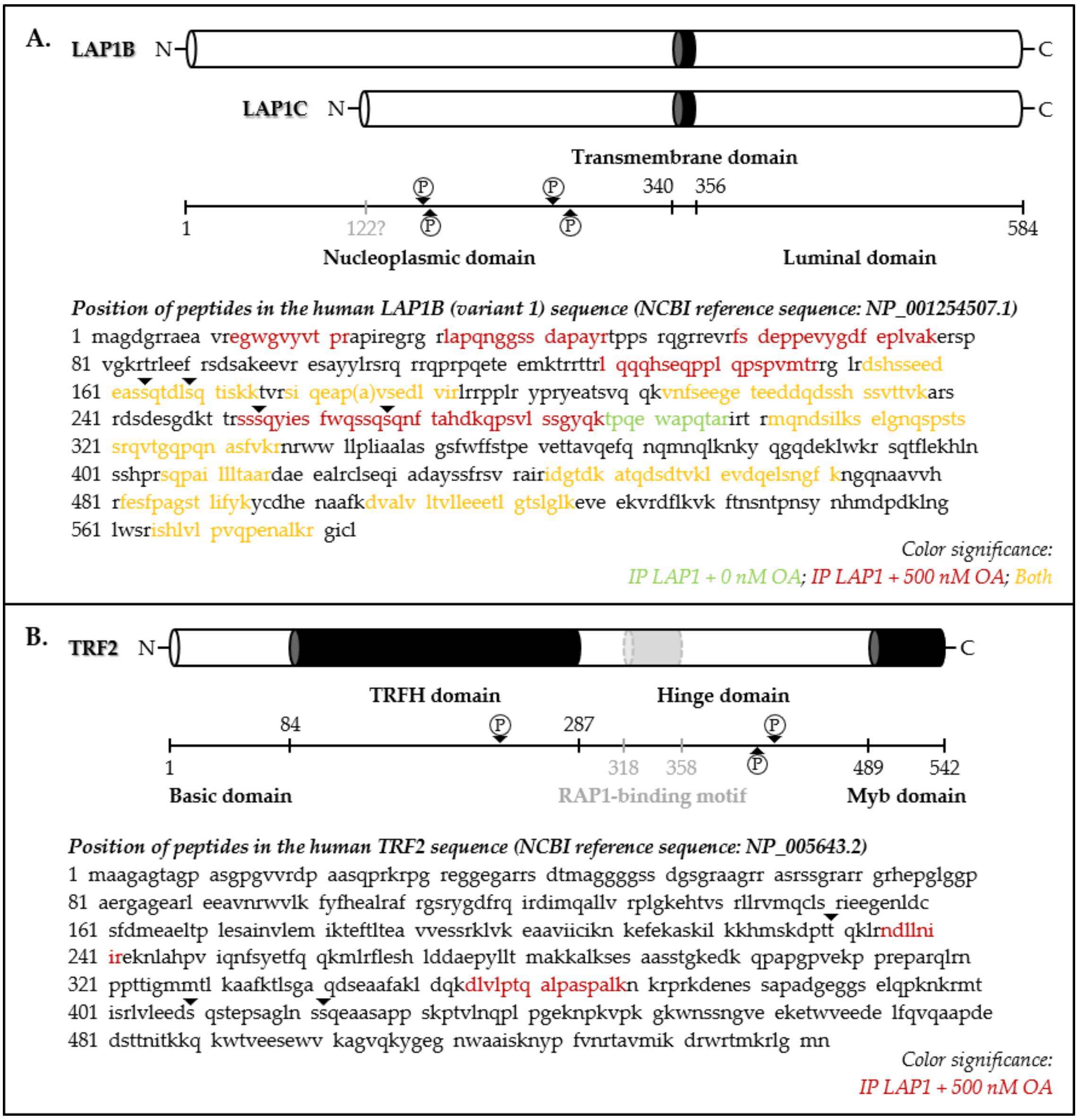

Supplementary Figure 3. Alignment of LAP1 and TRF2 peptides, identified by HPLC–MS after LAP1 co-immunoprecipitation in the human SH-SY5Y cell line, with the corresponding protein sequences. (**A**) Position of LAP1 peptides in the known sequence of human LAP1B variant 1 (NCBI reference sequence: NP_001254507.1 [49]). (**B**) Position of TRF2 peptides in the known human TRF2 sequence (NCBI reference sequence: NP_005643.2 [50]). In this experiment, SH-SY5Y cells' whole lysates were immunoprecipitated with Dynabeads Protein G-conjugated LAP1-specific antibody upon cell exposure to okadaic acid (500 nM for 3 h) or in normal conditions. Following SDS–PAGE, LAP1B (≈ 68 kDa) and LAP1C (≈ 56 kDa) protein bands were analyzed by HPLC–MS. Unique LAP1 peptides were identified, including six peptides in the condition of PP1/PP2A phosphatase activity inhibition alone (red), one peptide in the control condition alone (green) and 15 peptides in both conditions (orange) (A). Two unique TRF2 peptides were identified in the proximity of the 68 kDa protein band only in the okadaic acid treatment condition (red) (B). A schematic representation of the general structure of LAP1B, LAP1C (with a predicted translation initiation site at amino acid position 122 [19]) (A) and TRF2 (B) is shown. Arrowheads indicate DNA damage-regulated phosphorylation sites recognized by ATM/ATR in each protein [41,48]. ATM, ataxia-telangiectasia mutated protein; ATR, ATM- and Rad3-related protein; HPLC–MS, high-performance liquid chromatography–mass spectrometry; IP, immunoprecipitation; LAP1, lamina-associated polypeptide 1; OA, okadaic acid; PP1/2A, protein phosphatase 1/2A; RAP1, repressor/activator protein 1; SDS–PAGE, sodium dodecyl sulfate–polyacrylamide gel electrophoresis; TRF2, telomeric repeat-binding factor 2; TRFH, telomeric repeat-binding factor homology.

A.

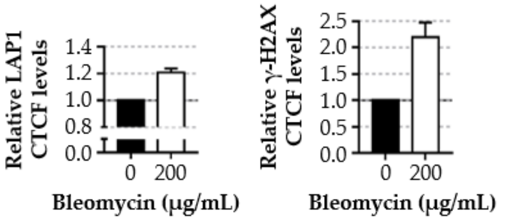

B.

Coefficient	Bleomycin (μg/mL)	
	**0**	**200**
**M_1 (γ-H2AX/LAP1)**	0.41 ± 0.01	0.44 ± 0.02
**M_2 (LAP1/γ-H2AX)**	0.40 ± 0.01	0.46 ± 0.02
**PCC**	0.49 ± 0.01	0.52 ± 0.02

C.

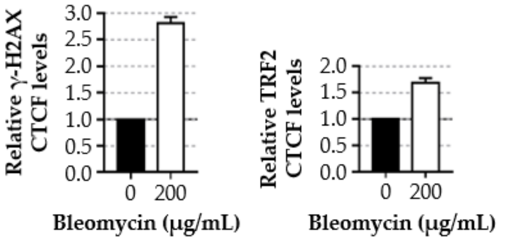

D.

Coefficient	Bleomycin (μg/mL)	
	**0**	**200**
**M_1 (TRF2/γ-H2AX)**	0.55 ± 0.01	0.66 ± 0.03
**M_2 (γ-H2AX/TRF2)**	0.75 ± 0.01	0.83 ± 0.02
**PCC**	0.74 ± 0.01	0.78 ± 0.02

E.

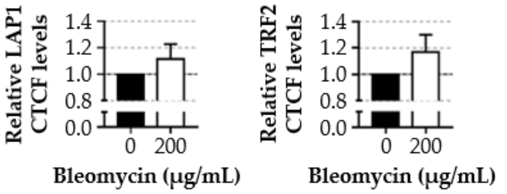

F.

Coefficient	Bleomycin (μg/mL)	
	**0**	**200**
**M_1 (TRF2/LAP1)**	0.57 ± 0.05	0.59 ± 0.01
**M_2 (LAP1/TRF2)**	0.66 ± 0.03	0.67 ± 0.02
**PCC**	0.70 ± 0.02	0.70 ± 0.02

Supplementary Figure 4. Analysis of co-localization between LAP1, TRF2 and γ-H2AX in the human HeLa cell line. (**A**, **C**, **E**) Relative LAP1/γ-H2AX (A), TRF2/γ-H2AX (C) and LAP1/TRF2 (E) CTCF levels in the nucleus [estimated in relation to untreated cells (0 μg/mL) using z-stacks] in control conditions and after DNA damage induction through cell exposure to 200 μg/mL of bleomycin for 30 min, followed by 6 h of recovery. Quantitative data are presented as mean ± SEM (*n* = 3, 30 (A, C) or 25 (E) cells per experiment). (**B**, **D**, **F**) Summary of spatial co-occurrence (M_1 and M_2) and intensity correlation (PCC) coefficients associated with nuclear LAP1/γ-H2AX (B), TRF2/γ-H2AX (D) and LAP1/TRF2 (F) co-localization analysis (using z-stacks) in the same experimental conditions specified in (A, C, E). Quantitative data are presented as mean ± SEM [*n* = 3, 30 (B, D) or 25 (F) cells per experiment]. In these assays, upon fixation, HeLa cells were immunostained with specific primary antibodies against LAP1, TRF2 and γ-H2AX linked to AF 488- or AF 594-conjugated secondary antibodies, followed by incubation with DAPI-containing mounting medium. Image acquisition was performed using an LSM 880 confocal laser scanning microscope with Airyscan. γ-H2AX, histone variant H2AX phosphorylated at Ser139; AF, Alexa Fluor; CTCF, corrected total cell fluorescence; DAPI, 4′,6-diamidino-2-phenylindole; LAP1, lamina-associated polypeptide 1; M_1, fraction of red fluorophore overlapping with green fluorophore; M_2, fraction of green fluorophore overlapping with red fluorophore; PCC, Pearson's correlation coefficient; SEM, standard error of the mean; TRF2, telomeric repeat-binding factor 2.
